# Phylogenomic analysis of trichomycterid catfishes (Teleostei: Siluriformes) inferred from ultraconserved elements

**DOI:** 10.1038/s41598-020-59519-w

**Published:** 2020-02-14

**Authors:** Luz E. Ochoa, Aléssio Datovo, Carlos DoNascimiento, Fabio F. Roxo, Mark H. Sabaj, Jonathan Chang, Bruno F. Melo, Gabriel S. C. Silva, Fausto Foresti, Michael Alfaro, Claudio Oliveira

**Affiliations:** 1 Departamento de Morfologia, Instituto de Biociências, Universidade Estadual Paulista, Botucatu, SP Brazil; 2 Museu de Zoologia da Universidade de São Paulo, São Paulo, SP Brazil; 3 Instituto de Investigación de Recursos Biológicos Alexander von Humboldt, Villa de Leyva, Boyacá, Colombia; 4 The Academy of Natural Sciences of Drexel University, Philadelphia, PA USA; 5 Department of Ecology and Evolutionary Biology, University of California, Los Angeles, CA USA

**Keywords:** Taxonomy, Ichthyology

## Abstract

The family Trichomycteridae is one of the most diverse groups of freshwater catfishes in South and Central America with eight subfamilies, 41 genera and more than 300 valid species. Its members are widely distributed throughout South America, reaching Costa Rica in Central America and are recognized by extraordinary anatomical specializations and trophic diversity. In order to assess the phylogenetic relationships of Trichomycteridae, we collected sequence data from ultraconserved elements (UCEs) of the genome from 141 specimens of Trichomycteridae and 12 outgroup species. We used a concatenated matrix to assess the phylogenetic relationships by Bayesian inference (BI) and maximum likelihood (ML) searches and a coalescent analysis of species trees. The results show a highly resolved phylogeny with broad agreement among the three distinct analyses, providing overwhelming support for the monophyletic status of subfamily Trichomycterinae including *Ituglanis* and *Scleronema*. Previous relationship hypotheses among subfamilies are strongly corroborated, such as the sister relationship between Copionodontinae and Trichogeninae forming a sister clade to the remaining trichomycterids and the intrafamilial clade TSVSG (Tridentinae-Stegophilinae-Vandelliinae-Sarcoglanidinae-Glanapteryginae). Monophyly of Glanapteryginae and Sarcoglanidinae was not supported and the enigmatic *Potamoglanis* is placed outside Tridentinae.

## Introduction

Unraveling the relationships of major sections of the Tree of Life is one of the most daunting challenges of the evolutionary biology. Massively parallel DNA sequencing (so-called Next-gen sequencing) is a promising tool that is helping to resolve the interrelationships of longstanding problematic taxa^1–4^. One of the most common classes of phylogenomic methods involves the sequence capture of nuclear regions in the flanks and cores of the ultraconserved elements (UCEs)^2^. The more variable flanking UCE regions allow a better resolution of nodes across a broad range of evolutionary timescales in a given phylogeny^2^. As variation in the flanks increases with distance from the core UCE, this combined approach displays a balance between having a high enough substitution rate while minimizing saturation, thus providing information for estimating phylogenies at multiple evolutionary timescales^2,3^. Recent studies of actinopterygians^5^, flatfishes^6^, cichlids^7^, ostariophysan^8^, acanthomorphs^9^, Loricariidae^10^, knifefishes^11^, among other vertebrates groups^3,12^, have shown that UCEs are excellent markers for phylogenetic studies because of their ubiquity among taxonomic groups^13^, low degrees of paralogy^14^, and low saturation^3^. According to Gilbert *et al*.^15^, the phylogenetic informativeness of the combined flank and core regions of UCEs outperfoms protein-coding genes used in multilocus studies. Additionally, phylogenomic approaches are characterized by their potential to collect data from at least one order of magnitude more loci than the traditional sequencing techniques applied to protein-coding legacy marker’s.

The present survey is the first to employ a new bait set to ostariophysans^16^ and high-throughput sequencing to address evolutionary relationships in the large catfish family Trichomycteridae (pencil and parasitic catfishes). The family contains 319 valid species^17^ characterized by a highly modified opercular system, with opercular and interopercular bones usually armed with distinct patches of sharp odontodes (integumentary teeth). Trichomycterids have one of the broadest ranges of trophic strategies known within a single catfish family, including insectivory, omnivory, carnivory, necrophagy, mucophagy, lepidophagy, and hematophagy^18–25^. The family has a wide distribution mostly in the Neotropical freshwater basins of Central and South America^26,27^ from Costa Rica to Chilean Patagonia, occurring on both versants of the Andes, and even in a few insular^27,28^ and caves environments with stygobiotic species.

Eight trichomycterid subfamilies are currently recognized: Copionodontinae, Glanapteryginae, Sarcoglanidinae, Stegophilinae, Trichogeninae, Trichomycterinae, Tridentinae, and Vandelliinae^20,29^. Only two papers have used explicit cladistic analyses to test the interrelationships among all eight subfamilies, one based on morphological data^29^ and the other based on nuclear and mitochondrial genes^30^. In spite of such recent advances, the descriptions of new species and clades based on labile morphological characters^31^ and hurried changes in subfamilial classification^32^ present obstacles to reconstructing the evolutionary history of Trichomycteridae. Here we present a phylogenomic analysis of a dataset of ultraconserved DNA elements (UCEs) and their flanking regions representing over 902 loci from 139 trichomycterid taxa (about 47% of species diversity of the family) to infer a new well -supported hypothesis of phylogenetic relationships discuss recent taxonomic changes in Trichomycteridae and provides an evolutionary framework to explore macroevolutionary and biogeographic processes that modeled the exceptional diversity in the Neotropics.

## Results

### Phylogenetic relationships within trichomycteridae

**Figure 1:**
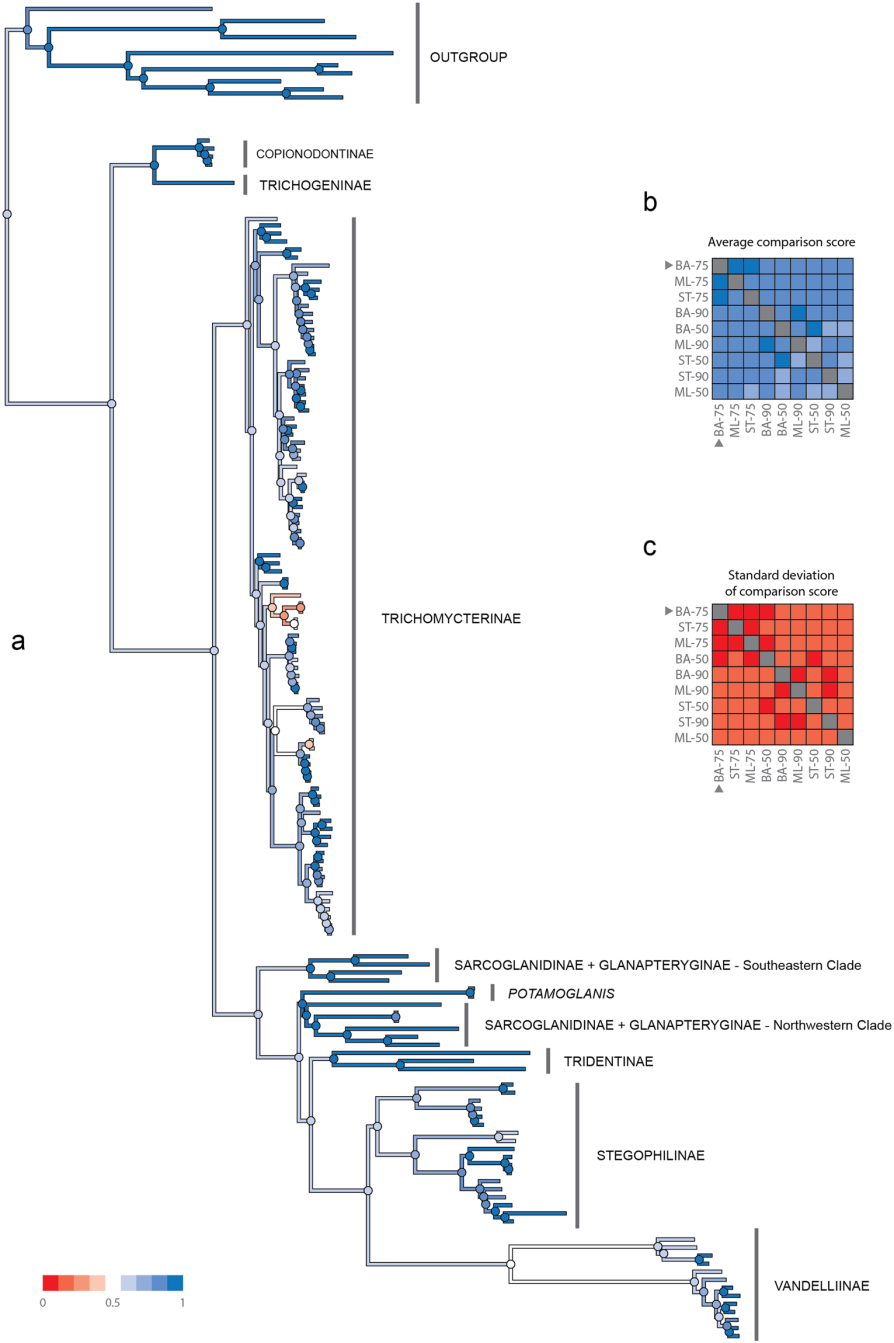
Pairwise comparison among all nine trees obtained by the distinct inference methods (ML, Bayesian and Species tree) with varied levels of data completeness (50, 75 and 90%). (**a**) highest values of global topological similarity (**b**) highest average and (**c**) lowest standard deviation of the element-based comparison score of Bremm *et al*. (2011).

**Figure 2:**
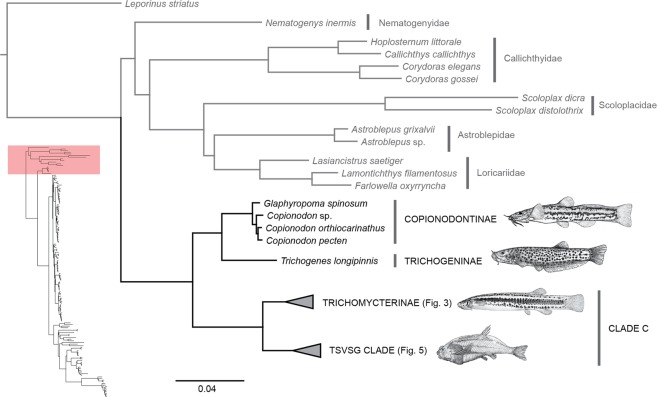
Phylogenetic hypothesis of Trichomycteridae from 902 ultraconserved loci using Bayesian analysis of concatenated data, highlighting the phylogenetic relationships for Copionodontinae -Trichogeninae. All nodes supported Bayesian posterior probabilities >0.99.

**Figure 3:**
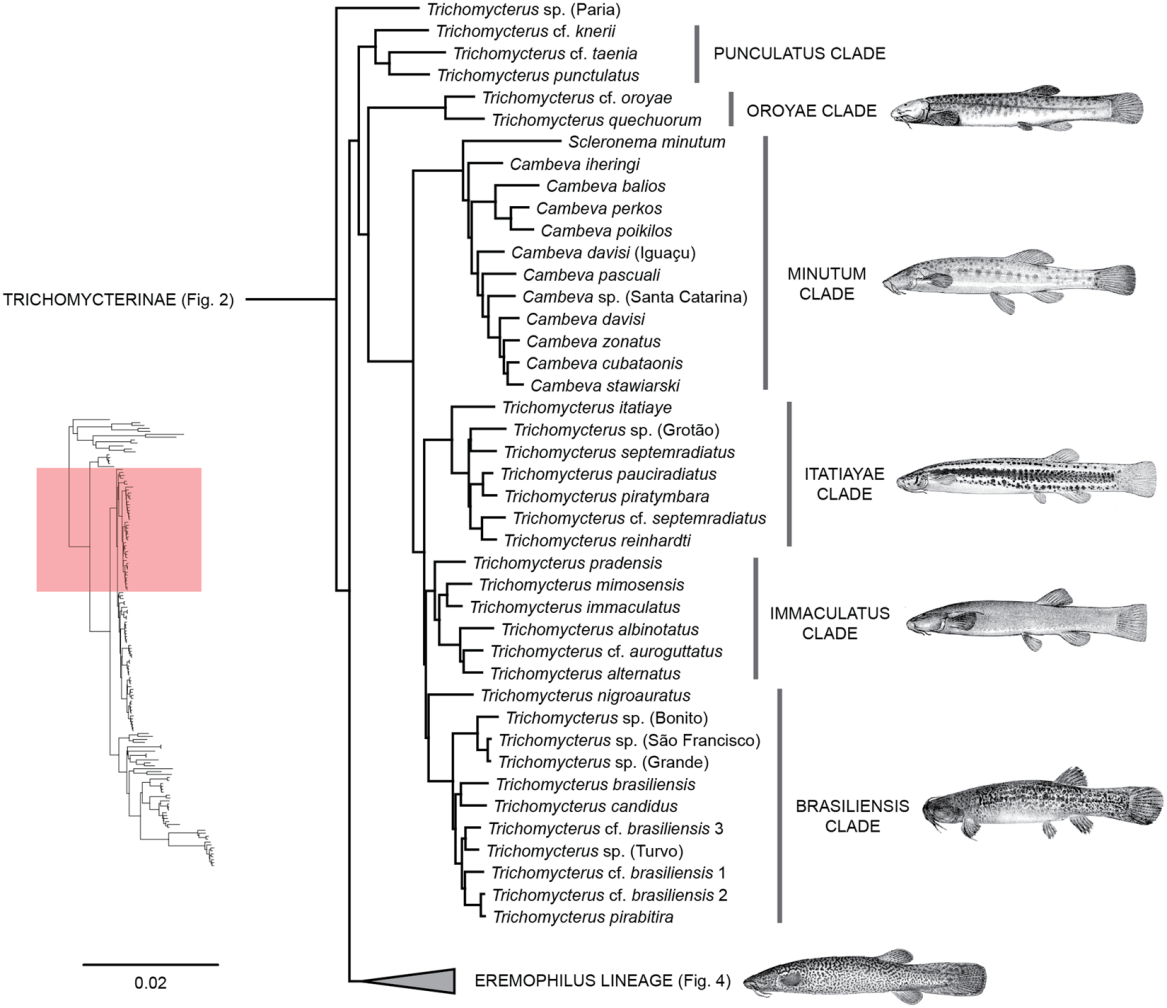
Phylogenetic hypothesis of Trichomycteridae from 902 ultraconserved loci using Bayesian analysis of concatenated data, highlighting the phylogenetic relationships for *Trichomycterus* lineage. All nodes supported Bayesian posterior probabilities >0.99.

**Figure 4:**
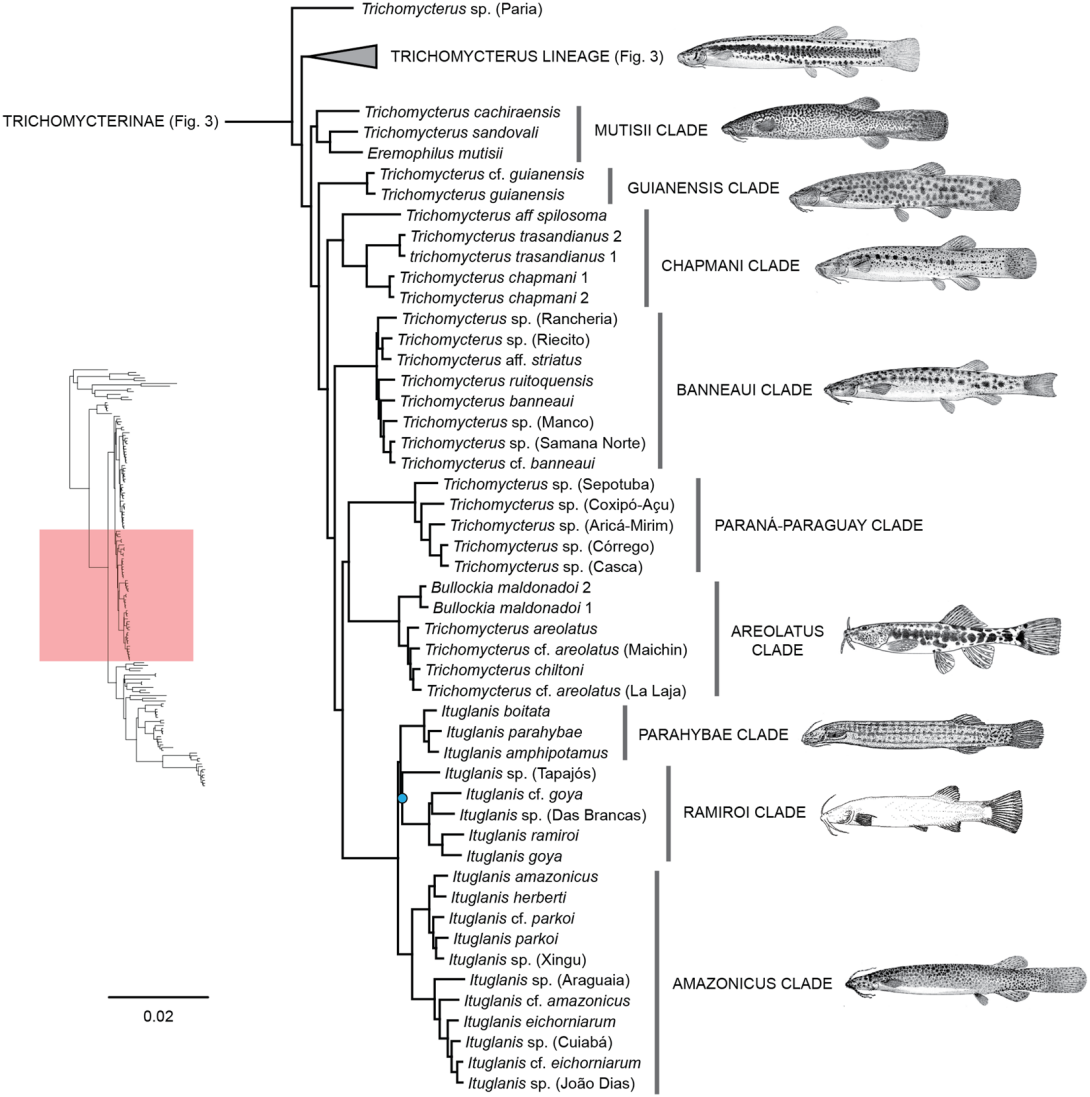
Phylogenetic hypothesis of Trichomycteridae from 902 ultraconserved loci using Bayesian analysis of concatenated data, highlighting the phylogenetic relationships for *Eremophilus* lineage. All nodes supported Bayesian posterior probabilities >0.99 and blue nodes with posterior probabilities <0.99.

**Figure 5:**
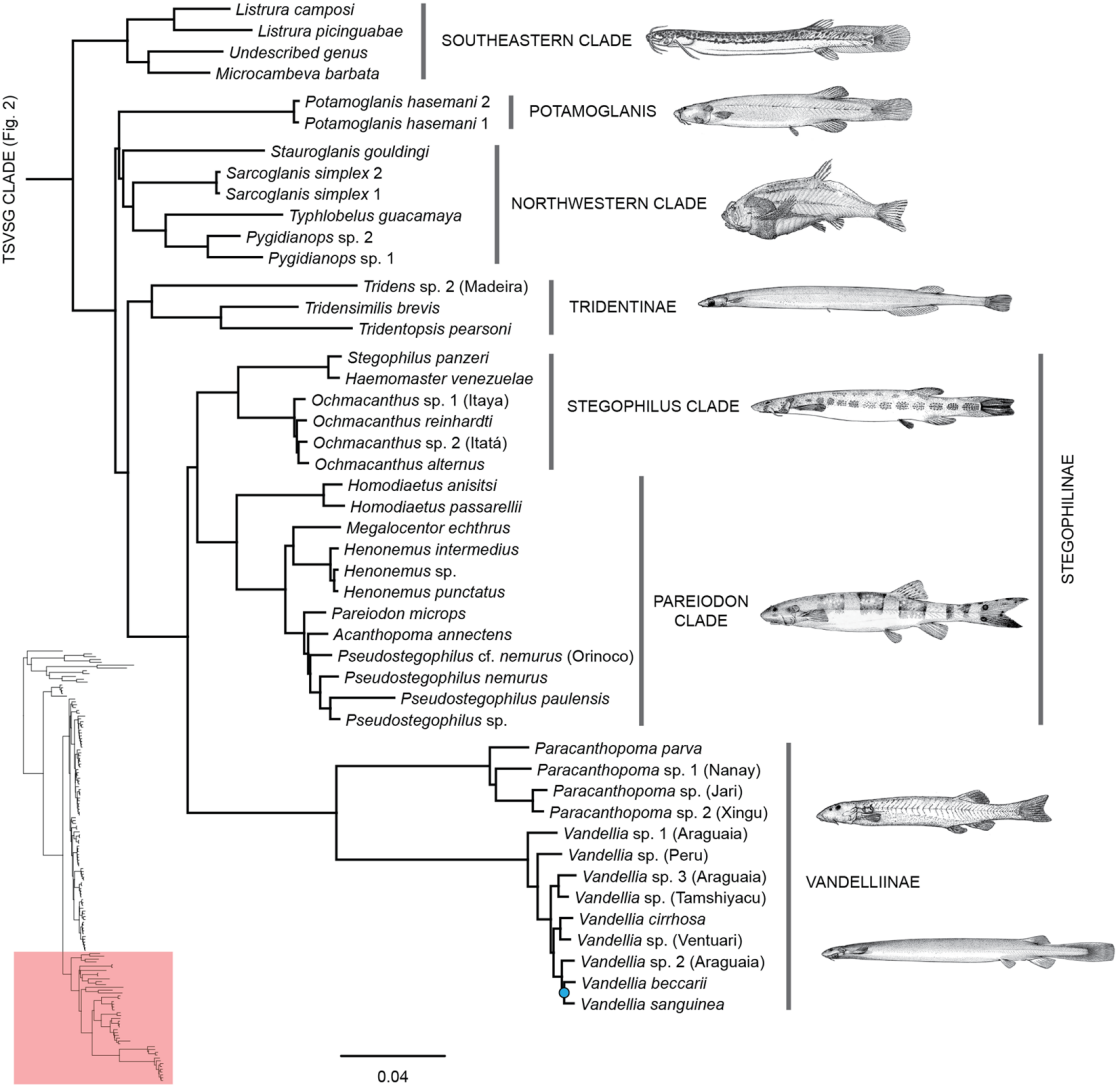
Phylogenetic hypothesis of Trichomycteridae from 902 ultraconserved loci using Bayesian analysis of concatenated data, highlighting the phylogenetic relationships for TSVSG clade. All nodes supported Bayesian posterior probabilities >0.99 and blue nodes with posterior probabilities <0.99.

The DNA sequencing yielded a total of 323 million reads with an average of 2.1 million reads per sample (range = 40,059–6.4 million). These reads were assembled into an average of 8,752 contigs per sample (95CI, min = 176, max = 36,085), having an average length of 597 bp (Supplementary Table 2). An average (per sample) of 1,321 of those contigs matched the UCE loci from the target capture probes used and the average length of UCE-matching contigs was 598 bp (range = 164–971). The size of each matrix according to their completeness level was 1,379 (50%), 902 (75%) and 432 (90%) loci. ML and Bayesian trees inferred from each locus alignment showed identical topologies. Using a method of species-tree analysis (ASTRAL) in which a species tree history is estimated from independent gene histories, we recovered species trees partially concordant with the concatenated analysis. The ASTRAL species trees for each matrix (50%, 75%, 90% completeness) were much less resolved and had lower support values than either the Bayesian or the ML tree estimated from the concatenated dataset. In a comparison of the species tree for the 75% matrix with the Bayesian tree, approximately 25 nodes of 153 species-tree nodes showed bootstrap values < 90%; however, the species tree recovered most relationships of the gene trees. Pairwise comparisons among all nine trees obtained by the distinct inference methods (ML, Bayesian, and Species tree) with varied levels of data completeness (50%, 75%, 90%) indicates that the Bayesian tree with 75% complete matrix (Fig. 1a) exhibits the highest values of global topological similarity. This is indicated by the highest average (Fig. 1b) and lowest standard deviation (Fig. 1c) of the element-based comparison score of Bremm *et al*.^33^. The Bayesian tree with 75% complete matrix is accordingly chosen as the reference tree for the present discussion of trichomycterid relationships. Figure 1a depicts the average conservation of the nodes of the reference tree against all other trees (Supplementary Figs. 1–8), indicating high and low levels of similarity with shades of blue and red, respectively. The detailed topology of the Bayesian tree with 75% complete matrix is represented in Figs. 2–5.

The 75% complete matrix contains 284,349 characters (including indels), 107,270 parsimony informative sites and 30.81% of missing data. The Bayesian (Figs. 2–5) and ML (Supplementary Fig. 4) analyses for this matrix returned identical topologies. Of the 152 nodes, 150 (98.68%) were highly supported in the Bayesian tree (>0.99PP), whereas a subset of 134 of those nodes (89.79%) were also highly supported in the ML tree (>75% bootstrap score). Two nodes (1.36%) showed low support in ML trees with 50% and 8% bootstrap values, respectively, but in the Bayesian tree just one node had a low posterior probability (p = 0.5999, in the relationships of *Ituglanis goya, Ituglanis ramiroi* and *Ituglanis* sp. (Tapajos) (Fig. 4).

The phylogeny supports with highest confidence (100% bootstrap; PP = 1) the monophyly of Trichomycteridae and the subfamilies Copionodontinae (100% of the genera sampled), Stegophilinae (81.8% of the genera sampled), Trichomycterinae *sensu* Datovo & Bockmann^29,30^, Tridentinae *sensu* Baskin^34^, (75% of the genera sampled) and Vandelliinae (50% of the genera sampled) (Figs. 2–5). Our analysis provides strong evidence for the monophyly of the Clade B *sensu* Datovo & Bockmann^29^, composed of Copionodontinae and Trichogeninae (100% bootstrap; PP = 1.0; Fig. 2). This clade is herein named the Trichogeninae-group and its placement as the sister group to all remaining trichomycterids is equally well-supported. The Copionodontinae and the genus *Copionodon* (*C*. *pecten, C*. *orthiocarinathus* and *Copionodon* sp.) are both supported as monophyletic.

Clade C was recovered with a basal dichotomy between the TSVSG clade (Tridentinae-Stegophilinae-Vandelliinae-Sarcoglanidinae-Glanapteryginae) and Trichomycterinae (Fig. 2). The phylogenomic data provide strong evidence for the monophyly of Trichomycterinae. Our definition of the subfamily includes *Ituglanis* and *Scleronema* along with the traditional trichomycterine genera, but excludes *Potamoglanis*. Our study confirms the non-monophyly of *Trichomycterus sensu lato*, the largest trichomycterid genus has 54% of the family’s diversity (Figs. 3 and 4). In our topology, an undescribed trichomycterine from Paria on the Caribbean coast of Venezuela (*Trichomycterus* sp. (Paria), is placed sister to all remaining trichomycterines (Fig. 3) which group into two major clades termed the *Trichomycterus* and *Eremophilus* lineages.

In the *Trichomycterus* lineage, two clades diverge early from the remaining taxa. The first one to diverge is the Punctulatus Clade composed of *T*. cf. *knerii* (Orinoco) and two species from Pacific coastal rivers draining the Central Andes, *T. punctulatus* and *T*. cf. *taenia*. The second one to diverge is the Oroyae Clade composed of *T*. cf. *oroyae* and *T. quechuorum*, two Andean species from the upper Amazonas Basin. Remaining taxa form a large clade that includes *Cambeva*, *Scleronema*, and all Brazilian species of *Trichomycterus*. The Minutum Clade contains *Scleronema minutum* and several species formerly placed in *Trichomycterus* and recently classified into the new genus *Cambeva* (*C*. *balios*, *C*. *cubataonis, C*. *davisi*, *C. iheringi, C*. *pascuali*, *C*. *perkos, C*. *poikilos*, *C*. *stawiarski* and *C*. *zonatus*). The Brazilian species of *Trichomycterus* are grouped into three main successive subclades. The first one includes (Itatiayae Clade) includes *T*. *itatiaye*, *T*. *pauciradiatus, T*. *piratymbara, T*. *reinhardti*, *T*. *septemradiatus*, *T*. cf. s*eptemradiatus* and *Trichomycterus*. sp. (Grotão). The second (Immaculatus Clade) includes *T. albinotatus*, *T. alternatus*, *T*. cf. *auroguttatus*, *T. immaculatus*, *T. mimosensis* and *T. pradensis*. The third one (Brasiliensis Clade) includes *T. brasiliensis*, *T*. cf. *brasiliensis*, *T. candidus*, *T*. *nigroauratus, T. pirabitira*, plus four undescribed species identified by sampling localities, *Trichomycterus* sp. (Grande), *Trichomycterus*. sp. (São Francisco), *Trichomycterus* sp. (Bonito), and *Trichomycterus* sp. (Turvo) (Brasiliensis Clade).

The second major clade in Trichomycterine lineage (*Eremophilus* lineage) exhibits four successive subclades at its base (Fig. 4). The first to split (Mutisii Clade) includes *T. cachiraensis, T. sandovali*, and the monotypic *Eremophilus mutisii*, all from the Magdalena basin. The next one (Guianensis Clade) joins *T. guianensis* and *T*. cf. *guianensis* from the Essequibo basin. The third (Chapmani Clade) includes *T*. *chapmani, T*. aff. *spilosoma*, and *T. transandianus*, each from separate trans-Andean drainages in Colombia. The fourth (Banneaui Clade) includes *T. banneaui*, *T*. aff. *striatus, T. ruitoquensis*, one undetermined species from Rancheria River, *Trichomycterus* sp. (Rancheria), and three undetermined species from the Magdalena basin, *Trichomycterus* sp. 4 (Riecito), *Trichomycterus* sp. (Manco) and *Trichomycterus* sp. (Samaná Norte). A group of five undescribed species of *Trichomycterus* from the Paraná-Paraguay basin forms a monophyletic group that is sister group to the Areolatus Clade, composed of *Bullockia maldonadoi*, *T. areolatus*, and *T. chiltoni*. Finally, the monophyletic *Ituglanis* occupies the apex of this major trichomycterine lineage. The species sampled grouped into two main clades. One contains the Parahybae Clade (*I. boitata*, *I. parahybae*, and *I. amphipotamus*) and Ramiroi Clade composed of *I. goya*, *I*. cf. *goya*, and *I. ramiroi* and two undescribed Amazonian species, *Ituglanis* sp. (Das Brancas) and *Ituglanis* sp. (Tapajos). The second one (Amazonicus clade) combines species from the Amazon and La Plata systems, with a subclade composed of *I. amazonicus*, *I. herberti*, *I*. cf. *parkoi*, *Ituglanis*. sp. (Xingu) and *I. parkoi* sister to a second subclade including *I*. cf. *amazonicus*, *I. eichhorniarum, I*. cf. *eichhorniarum* plus three undescribed species *Ituglanis*. sp. (Araguaia), *Ituglanis*. sp. (Cuiabá) and *Ituglanis*. sp. (João Dias).

The TSVSG Clade includes the subfamilies Tridentinae, Stegophilinae, Vandelliinae, Sarcoglanidinae, and Glanapteryginae (Fig. 5). We analyzed five representatives of three currently recognized glanapterygine genera, including the most generalized *Listrura* (*L. camposi* and *L. picinguabae*) and the highly derived psammophilic *Pygidianops* (*Pygidianops* sp. 1, and *Pygidianops* sp. 2) and *Typhlobelus* (*T. guacamaya*). The only glanapterygine genus not analyzed was *Glanapteryx* which contains two nominal species. Half of the sarcoglanidine genera were analyzed (*Microcambeva barbata*, *Sarcoglanis simplex* and *Stauroglanis gouldingi*); missing were *Ammoglanis*, *Malacoglanis*, and *Stenolicmus*. An undescribed taxon apparently belonging to the Glanapteryginae-group, Trichomycteridae n. gen. (de Pinna & Datovo; pers. comm.) also was incorporated into our study.

The resulting hypothesis for the TSVSG Clade did not support the monophyly of Glanapteryginae and Sarcoglanidinae, respectively, or the sister group relationship between those subfamilies. Rather, representatives of both subfamilies from Atlantic coastal drainages grouped into one clade (Southeastern Clade), while those from the Amazon and Orinoco basins (Northwestern Clade) and the enigmatic *Potamoglanis* from the Amazon and Paraguay basins formed a second clade more closely related to other TSVSG taxa. The Southeastern Clade is at the base of the TSVSG clade and includes the undescribed genus of de Pinna & Datovo, *Microcambeva barbata* (Sarcoglanidinae), and the two species of *Listrura* (Glanapteryginae). *Potamoglanis* is sister to the Northwestern Clade composed of *Sarcoglanis simplex, Stauroglanis gouldingi, Typhlobelus guacamaya* and the two species of *Pygidianops*. That clade is the sister to the so-called Vandelliinae-group, a node with strong support that includes the Tridentinae (*sensu stricto*), Stegophilinae, and Vandelliinae. Tridentinae (minus *Potamoglanis*) is at the base of the whole group with Stegophilinae and Vandelliinae appearing as sister taxa. Our results do not support the recent allocation of *Potamoglanis hasemani* in the Tridentinae (*contra*; Henschel *et al*. 2017, see Discussion) and resolve *Tridens* sp. 2 (Madeira) as the sister group to the clade formed by *Tridensimilis brevis* and *Tridentopsis pearsoni*. All SH tests using the 75% complete UCE matrix showed that topologies supporting the monophyly of the Glanapteryginae and the Sarcoglanidinae, respectively, their mutual sister-group relationships, as well as the alternative hypotheses of relationships of *Potamoglanis*^32,35,36^ were significantly worse (P < 0.001) than the recovered phylogeny (Table 1).

**Table 1:** Results from the SH test estimated from UCEs dataset showing the likelihood (LH), difference in likelihood (D[LH]), standard deviation (SD), p-values, Bonferroni correction of *p* values and false discovery rate (FDR) *p* values for each hypothesis of Trichomycteridae relationships, tested against the best tree from the RAxML analysis.

Hypothesis	Likelihood (LH)	D(LH)	SD	5%	2%	1%	*p*-values	Corrected *p*-values	Corrected *p*-values (FDR)
ML best tree	−2807843.36								
Glanapteryginae and Sarcoglanidinae monophyletic at position of currently hypothesis	−2828166.17	−20322.80	341.94	yes	yes	yes	0	0	0
(Glanapteryginae + Sarcoglanidinae) + (Tridentinae, (Stegophilinae + Vandelliinae))	−2828763.44	−20920.07	360.58	yes	yes	yes	0	0	0
Potamoglanis + (Tridens + (Tridensimilis + Tridentopsis))	−2808510.61	−667.25	76.64	yes	yes	yes	0	0	0
Potamoglanis + (Tridens + (Stegophilinae + Vandelliinae))	−2808064.34	−220.97	59.77	yes	yes	yes	0.0002	0.0013	0.0002
Glanapteryginae monophyletic derived position Pygidianops + Typhlobelus	−2819603.80	−11760.44	213.07	yes	yes	yes	0	0	0
Sarcoglanidinae basal position Microcambeva	−2839796.53	−31953.16	415.15	yes	yes	yes	0	0	0

With nine of 11 genera of the Stegophilinae represented in our analysis (only *Apomatoceros* and *Schultzichthys* are missing), the internal relationships of the subfamily were well resolved and mostly in agreement with a recent morphological revision^36^. Our hypothesis divides the Stegophilinae into two major subgroups. The largest clade (*Pareiodon* Clade) contains *Homodiaetus* (*Ho. anisitsi* and *Ho. passarellii*) at the base and two subclades: one composed of the monotypic *Megalocentor echthrus* and *Henonemus* (*He. intermedius, He. punctatus*, and *Henonemus* sp.) and the second by the monotypic *Pareiodon* sister to monotypic *Acanthopoma* sister group to *Pseudostegophilus*. A few analyses do not support the monophyly of *Pseudostegophilus* (Supplementary Figs. 6–8). The second major stegophiline group (*Stegophilus* Clade) clusters *Ochmacanthus* (*O. alternus, O. reinhardti*, and two unnamed species of *Ochmacanthus* from the Itaya and Itatá Rivers) as sister group to the clade composed of *Stegophilus panzeri* and the monotypic *Haemomaster venezuelae*. In the Bayesian and ML analyses with 90% complete matrices the monophyly of the Stegophilinae is not recovered (Supplementary Figs. 2 and 5) and the resulting lineage, *Stegophilus* Clade + Vandelliinae shows extremely low support (<0.5 posterior probability and 50% bootstrap).

Two of the four vandelliine genera were included in our analysis, *Paracanthopoma* and *Vandellia*. The monophyly of each genus and the whole subfamily is strongly supported, but several species-level interrelationships showed low support (Fig. 5). Some minor differences were observed in the interspecific relationships of *Vandellia* across the different analyses (Supplementary Figs. 1, 3 and 8).

## Discussion

The present phylogenomic analysis is the largest molecular dataset ever assembled for Trichomycteridae. Interfamilial and intergeneric relationships are mostly congruent with previous hypotheses based on morphology^20,29,34,37^ and multilocus datasets^21,30,32^. The species tree was less resolved with low bootstrap values and switched the positions of some taxa compared to topologies supported by analyses of the concatenated dataset. Inconsistencies between trees^38,39^ can be caused by different factors such as gene duplication^40^ horizontal transfer^41^, and incomplete sorting of ancestral polymorphism^42–44,45^. Discrepancies in bootstrap values are associated with the traditional concatenation approach, insufficient data^46–48^ and issues inherent to multilocus bootstrapping methods^49,50^. However, the most significant reason why the species have different relationships amongst species trees should be attributable to deep coalescence processes, where multiples lineages tend to persist into the deeper portion of the species tree^51^. This pattern is common in groups with rapid and/or recent diversification, where the species tree are characterized by short branches^52^. In this case, gene lineages persist through the species trees and coalesce with gene lineages that are not from the most closely related species due to the short evolutionary time that is not sufficient for the fixation of gene lineages by genetic drift^49^.

Notwithstanding those limitations, our phylogenomic hypothesis supports the monophyly of Trichomycteridae. This result is congruent with all morphological studies, which provide a high number of unequivocal synapomorphies for the family^20,29,34,37^. Additionally, our topology obtained supports the monophyly and recognition of most clades previously recognized within the Trichomycteridae. For instance, Copionodontinae and Trichogeninae have been considered basal lineages since the first phylogenetic studies incorporating those taxa^20^. Members of these subfamilies show several plesiomorphic character-states not present in the remaining trichomycterids and the sister group relationship between Copionodontinae and Trichogeninae has been evidenced in other studies^20,29,37^.

**Figure 6:**
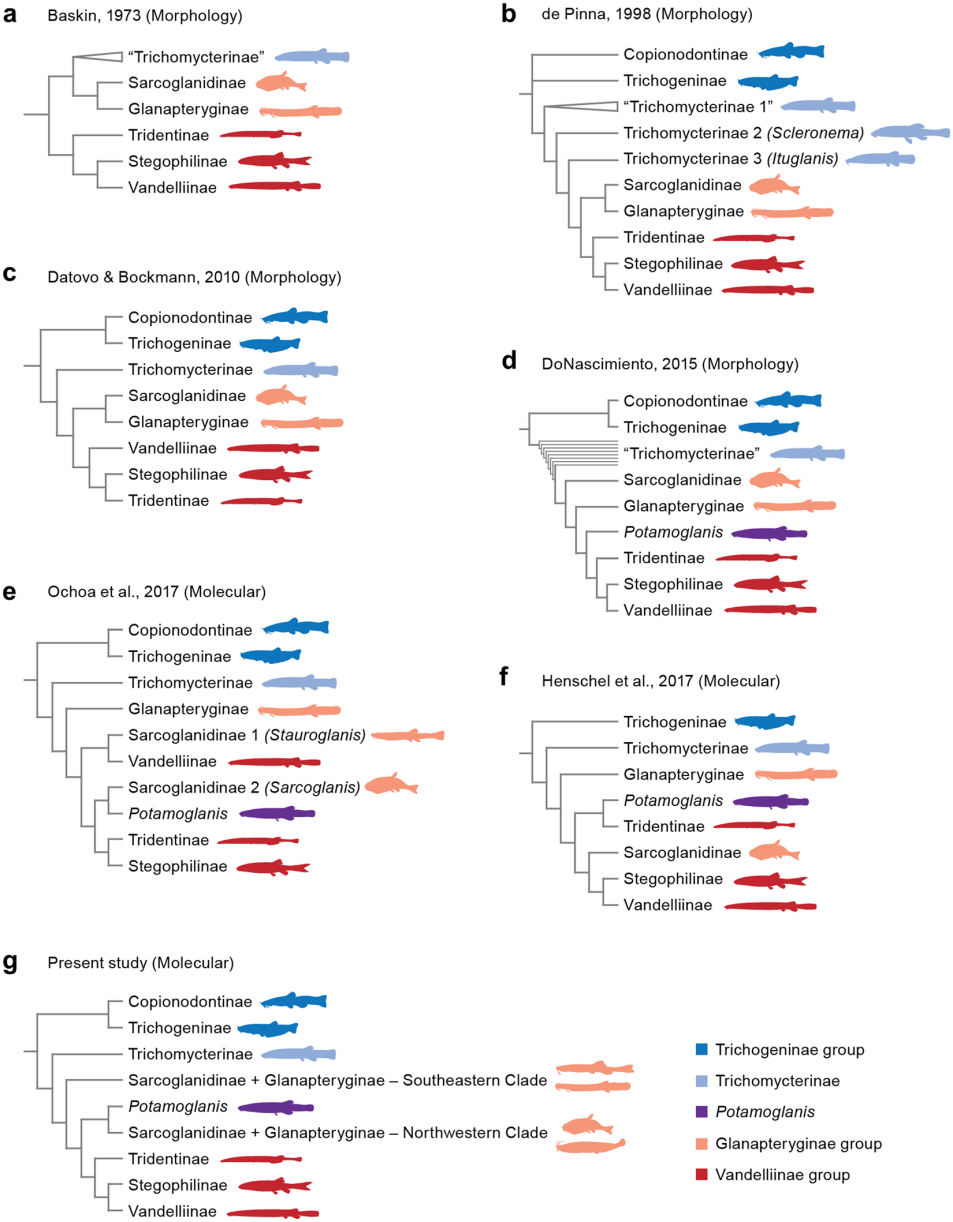
Phylogenetic hypothesis for Trichomycteridae.

Our results support the monophyly of the Clade C taxa that corresponds to the classic definition of the family, that is, the Trichomycteridae prior to the discoveries of trichogenines and copionodontines in the late 20^th^ century^37,53^. In the present analysis, Clade C is basally divided into two lineages: TSVSG clade and Trichomycterinae *sensu* Datovo & Bockmann^29^. Monophyly of TSVSG clade composed of the Tridentinae, Stegophilinae, Vandelliinae, Sarcoglanidinae, and Glanapteryginae was first proposed by Costa & Bockmann^54^ and corroborated by all subsequent morphological^20,29,36,55^ and molecular studies^30,32^. The major taxonomic change in the TSVSG clade was the formal incorporation of *Potamoglanis*, formerly referred to as the *Trichomycterus hasemani-*group. This group was long proposed to be related to the TSVSG clade, but its closer affinities are contentious. De Pinna^56^ was the first to draw attention to the presence of some highly derived features of *Potamoglanis hasemani*, such as its small body size and the presence of a single enormous cranial fontanel. These characters, although not unique, are shared with tridentines and de Pinna^35^ suggested the inclusion of *Potamoglanis* in that subfamily. The cladistic analysis of DoNascimiento^36^, with 49 terminal taxa and 520 morphological characters, refuted de Pinna’s^35^ hypothesis and supported *Potamoglanis* as the sister group of the clade comprising tridentines, stegophilines, and vandelliines (Fig. 6d). In the molecular study of Ochoa *et al*.^30^, *Potamoglanis* was the sister group to *Sarcoglanis* (Fig. 6e). More recently, Henschel *et al*.^32^ performed a multilocus analysis proposing *Potamoglanis* as sister group to tridentines (Fig. 6f). Based on that finding and ignoring prior hypotheses^30,36^, Henschel *et al*.^32^ formally erected the genus *Potamoglanis* as a member of an expanded Tridentinae. Nevertheless, the present analysis obtained a result similar to that of Ochoa *et al*.^30^ by grouping *Potamoglanis* with the sarcoglanidines and glanapterygines from the Amazon and Orinoco basins. It is worth mentioning that *Potamoglanis* does share some anatomical features traditionally considered to be synapomorphic at different levels of the Glanapteryginae-group, such as the possession of a toothless lateral process on the premaxilla and a long anterior process on the hyomandibula^32,57^. Moreover, all three synapomorphies proposed by Henschel *et al*.^32^ to support *Potamoglanis* as a tridentine are blatantly problematic as they are shared by some sarcoglanidines and glanapterygines. The first synapomorphy, expanded single cranial fontanel, is present in most members of the basal-most sarcoglanidine genus *Ammoglanis* (pers. obs.^58,59^). The second synapormophy, origin of the dorsal fin just above or posterior to the anal-fin origin, is absent in *P. anhanga*^57^ and present in *Listrura picinguabae*^60^ and some *L. costai*^61^ and not comparable in most glanapterygines, which lack a dorsal fin. The third proposed synapomorphy for *Potamoglanis* is the short ventral process of the opercle (p. 7^32^), however *Potamoglanis* has a process comparable in size or larger^32,57^ than that observed in several sarcoglanidines and glanapterygines (pers. obs.; see also *Stauroglanis gouldingi*^62^, *Glanapteryx anguilla*^35^; *Microcambeva barbata*^55^; *Ammoglanis pulex*^59^; *Microcambeva draco*^63^; *Pygidianops amphioxus*^64^ and several species of *Typhlobelus*^65^). In light of these issues and the conflicting hypotheses of relationships for *Potamoglanis* with or within the TSVSG clade, we consider the assignment of *Potamoglanis* to any trichomycterid subfamily impetuous. Therefore, for the sake of nomenclatural stability, the present paper follows the traditional concept of the Tridentinae, *i.e*. not including *Potamoglanis*^20,34^.

There has been some debate on the relationships of the Sarcoglanidinae and Glanapteryginae. The two subfamilies are traditionally considered sister taxa by most anatomical studies^29,34,55,66^ (Fig. 6a–c). However, the most recent morphological analysis^36^ and all molecular phylogenies of the Trichomycteridae^30,32^ refuted the monophyly of the Glanapteryginae-group (Fig. 6d–g). Based on morphology, DoNascimiento^36^ resolved the Sarcoglanidinae and Glanapteryginae as successive sister taxa to the Vandelliinae-group. Based on molecules Ochoa *et al*.^30^ placed Glanapteryginae at the base of the TSVSG clade; however, Sarcoglanidinae was non-monophyletic, with *Stauroglanis* closer to vandelliines and *Sarcoglanis* closer to *Potamoglanis*. In the topology of Henschel *et al*.^32^, tridentines plus *Potamoglanis* are intercalated between Glanapteryginae (at the base of the TSVSG clade) and the clade (Sarcoglanidinae + (Stegophilinae + Vandelliinae)). Our analysis shows even more striking result in which neither Glanapteryginae nor Sarcoglanidinae are monophyletic. Members of both subfamilies are clustered into two clades that are successive sister taxa to the Vandelliinae-group. Interestingly, our analysis groups the glanapterygines and sarcoglanidines from the Atlantic coastal drainages (*Listrura*, *Microcambeva*, and Trichomycteridae n. gen.) into one clade (Southeastern Clade), and members of both subfamilies from the Amazon and Orinoco basins (*Pygidianops*, *Sarcoglanis, Stauroglanis* and *Typhlobelus*) along with *Potamoglanis* (Amazon and Paraguay basins) into a second clade (Northwestern Clade). The glanapterygines and sarcoglanidines grouped in the latter clade curiously share several reductive features, such as extreme reductions in the pigmentation and numbers of opercular odontodes, interopercular odontodes, and premaxillary teeth (pers. obs.^58,62^).

Dismantling the Glanapteryginae-group is a drastic change that obviously demands further investigation, but this result is not so unexpected. A critical appraisal of the osteological characters listed to support various nodes of the Glanapteryginae-group demonstrates that several putative synapomorphies are highly homoplastic or vaguely delimited. For instance, the quadrate with a “posteriorly directed process” (p. 725)^55^ is considered a synapomorphy for the clade Glanapteryginae + Sarcoglanidinae^54,55^, but a large number of its members obviously lack this feature (pers. obs.; *Glanapteryx anguilla*^62^; *Ammoglanis pulex*^59^; *Microcambeva ribeira*e^67^; *M*. *draco*^63^; *Pygidianops amphioxus*^68^; and several species of *Typhlobelus*^65^). A second putative synapomorphy for the Glanapteryginae-group, hyomandibula with a long anterior process, is also problematic. The feature is also present in *Potamoglanis* and vandelliines^59^. Moreover, diagnoses and interrelationships among the putative basal-most genera of the Glanapteryginae and Sarcoglanidinae are particularly unstable and the limits of each subfamily are increasingly blurry^69^. For instance, new data suggest that *Ammoglanis pulex* is actually a glanapterygine, rather than a sarcoglanidine as originally described^70^. Allocation of newly discovered taxa (e.g., trichomycterid n. gen.) into one or another subfamily is often difficult and possibly arbitrary (pers. obs.^70^). These examples indicate the critical need for a taxonomically comprehensive revisionary study of the Glanapteryginae and Sarcoglanidinae, ideally combining morphological and molecular data.

Within the TSVSG clade, all morphological studies have postulated the existence of a monophyletic lineage consisting of Tridentinae, Stegophilinae, and Vandelliinae^20,29,34,36^ (Fig. 6a–d). This lineage, the Vandelliinae-group, is strongly supported by a profusion of anatomical specializations. Previous molecular analyses failed to recover the monophyly of the Vandelliinae-group and presented conflicting topologies within the TSVSG clade^30,32^ (Fig. 6e,f). The present study resolves a monophyletic Vandelliinae-group (Fig. 6g). Our tree also supports the Tridentinae as sister group to the clade Stegophilinae + Vandelliinae, thus agreeing with the topologies obtained by most previous analyses^20,32,34,36^.

Only Baskin^34^ tested the interrelationships among all four genera traditionally assigned to Tridentinae. In that morphological analysis, *Miuroglanis* and *Tridentopsis* are placed as the successive sister taxa to the node *Tridens + Tridensimilis*. As subsequent analyses never sampled all tridentine genera, Baskin’s^34^ scheme prevailed as the only cladistic study among tridentines for over 45 years. The recent analyses of DoNascimiento^36^ and Henschel *et al*.^32^, did not include *Miuroglanis* and *Tridentopsis*, respectively. Nevertheless, both analyses obtained a topology compatible with that of Baskin^34^. Unfortunately, we could not sample *Miuroglanis* and our resolution of tridentines differs from the past analyses by obtaining *Tridensimilis* as sister group to the clade *Tridens* + *Tridentopsis*. Our topology should be seen as provisional inasmuch the identification of “*Tridens* sp. Madeira” is inconclusive. That species does not perfectly fit the traditional definition of the genus and seems to exhibit morphological conditions apparently intermediate between *Tridens* and *Tridensimilis*.

Our hypothesis of interrelationships among stegophilines is almost identical to the comprehensive revision of the subfamily published by DoNascimiento^36^. In both analyses, Stegophilinae has a basal dichotomy into a clade clustering *Haemomaster, Ochmacanthus*, and *Stegophilus* and another grouping all remaining genera. The only difference between the two topologies is the placement of the monotypic *Acanthopoma* as sister group to *Pseudostegophilus* in our analysis. DoNascimiento’s^36^ tree placed *Acanthopoma* in a basal polytomy with *Pareiodon* and *Pseudostegophilus*. In the pioneering molecular analysis of the Vandelliinae-group, Fernandez & Schaefer^21^ obtained a sister -group relationship between *Acanthopoma* and *Pareiodon* in a rather distal position within Stegophilinae, as sister group to the clade (*Pseudostegophilus* (*Apomatoceros* + *Henonemus*)).

Different studies arrived at conflicting hypotheses of relationships among the vandelliine genera^29,32,34,36^. Our analysis includes representatives of *Paracanthopoma* and *Vandellia* and support the monophyly of each genus. However, proper resolution of the Vandelliinae will depends of a broad taxonomic revision of the group, since only a small fraction of its true diversity has been carefully studied and several species and genera await formal descriptions.

Among trichomycterids, controversy has surrounded the phyletic status and composition of the Trichomycterinae. Most traditional studies considered the subfamily to be a non-monophyletic assemblage that includes some taxa more closely related to the TSVSG clade than to other trichomycterines (Fig. 6a,b,d). Three trichomycterine subgroups were explicitly proposed as aligned with the TSVSG clade: *Scleronema*, *Ituglanis*, and *Potamoglanis* (previously referred to as *Trichomycterus hasemani* group). The inclusion of the lattermost genus within the TSVSG clade is unequivocal on both morphological^20,29,36,56^ and molecular grounds^30,32^. On the other hand, a *Scleronema-Ituglanis-*TSVSG relationship was rejected by Datovo & Bockmann^29^, who additionally provided morphological evidence for the grouping of these genera with the remaining trichomycterines (excluding *Potamoglanis*) into a monophyletic Trichomycterinae (Fig. 6c). This hypothesis was subsequently corroborated by all molecular analyses^30,32^, including the present one (Clade D; Fig. 6e,g).

Interrelationships within Trichomycterinae have never been extensively surveyed using morphological data, notwithstanding some proposals of small putative subgroups with restricted geographic distributions^20,71–76^. The multilocus analysis of Ochoa *et al*.^30^ was the first study to employ a substantial taxonomic sampling of the subfamily. That topology divided the Trichomycterinae into two major lineages and six main subclades (D1, D2, D3, D4, D5, and E). The present UCE analysis expands the previous sampling of trichomycterines by roughly 15% and the resulting subfamily tree exhibits only three most significant divergences from Ochoa *et al*.^30^ the placement of an undescribed trichomycterine from the Caribbean coast of Venezuela (previously unsampled) at the base of the whole subfamily and the non-monophyly of Clades D1 and D2. As in Ochoa *et al*.^30^, most trichomycterines are divided into two major clades: the herein termed *Eremophilus* lineage and the *Trichomycterus* lineage.

Within the *Eremophilus* lineage, a novel sister group relationship between *Ituglanis* and the clade comprising *Bullockia* plus the Chilean species of *Trichomycterus* is consistently supported, either by multilocus analyses^30^ and the present genomic study. Monophyly of the clade consisting of *Bullockia* and the Chilean species of *Trichomycterus* is also congruent between both molecular analyses and was likewise recovered in the morphological study by DoNascimiento^36^.

In the *Trichomycterus* lineage, the sister group relationship between *Scleronema* and a clade of species traditionally assigned to *Trichomycterus* and transferred to the recently erected genus *Cambeva* was originally proposed by Ochoa *et al*.^30^ and is also recovered herein. Recognition of a monophyletic subset of species of *Cambeva* (originally comprising *C. crassicaudatus*, *C. igobi*, and *C. stawiarski*), was first advanced by Wosiacki & de Pinna^74,77^, based on shared derived characters of the caudal skeleton. Subsequently, Datovo & Bockmann^29^ provided evidence from the head musculature supporting a clade consisting of *C. davisi* and *C. stawiarski*. Datovo *et al*.^78^ included *C. perkos* in that group based on the shared presence of a high number of branchiostegal rays and, more recently, Terán *et al*.^79^ found morphological support to include in that group also their newly described *C. ytororo*. Katz *et al*.^31^ diagnosed *Cambeva* based on a combination of plesiomorphic or general character states and some derived characters uniquely shared by *Cambeva* and *Scleronema*.

A notable finding of the present genomic analysis is consistent support for several subclades within *Trichomycterus* previously identified in multilocus analyses^30^. These subclades coincide with circumscribed geographic regions that had a denser taxonomic sampling in both multilocus and UCE analyses. One of these main subclades includes species from South and Southeastern Brazil and is subdivided into multiple lineages that are partially congruent with species groups proposed by morphology. Thus, the clade including *T. pauciradiatus*, *T. piratymbara*, *T. reinhardti*, and *T. septemradiatus*, was originally proposed as containing *T. reinhardti* and *T. pauciradiatus* and defined by the shared color pattern consisting of a broad dark brown stripe along the lateral midline, bordered above by a light yellow longitudinal zone^80^. Composition of this clade was later expanded to include *T. piratymbara* and *T. septemradiatus*^31^. However, some emblematic species groups within *Trichomycterus* have not been recovered herein or in previous molecular analyses, such as the *T. brasiliensis* species-complex that has transited through multiple definitions and main changes in its taxonomic composition^73,81,82^. A major effort is needed to increase the taxonomic sampling of *Trichomycterus* from the Andes and Guiana shield regions, whose few representative species analyzed in Ochoa *et al*.^30^ were found to have shifting positions in the present analyses, even jumping to different main clades (*e.g*. *T*. cf. *kneri* and *T. punctulatus*). Ideally, increased taxonomic representation in the molecular analyses should be accompanied by a comprehensive morphological survey in order to provide phylogenetic diagnoses for each of these remnant groups of *Trichomycterus*.

As mentioned earlier, Trichomycterinae concentrates most of the species-level diversity and most of the taxonomic problems inherent to the family. The identification of trichomycterine subgroups is necessary to advance in the systematics of the Trichomycteridae, but should avoid the premature and unreasonable proposition of new taxa. The recent erection of the genus *Cambeva*^31^ is a prime example. Ochoa *et al*.^30^ obtained a fully resolved trichomycterine phylogeny, but prudently concluded that taxonomic changes at moment were premature. Among the original results of that study, was a clade composed of nine species of *Trichomycterus* from southeastern Brazil that appeared as the sister group of *Scleronema*. One year later, Katz *et al*.^31^ transferred these species of *Trichomycterus* to a new genus, *Cambeva*, based on a molecular matrix, including only nine sequences of the 25 species allocated in *Cambeva*. Most, if not all, osteological characters proposed by Katz *et al*.^31^ to diagnose *Cambeva* and the clade *Cambeva* + *Scleronema* are incorrect as revealed by a test survey of the literature (e.g.)^73,83–86^ and examination of a few clear and stained specimens (AD, pers. obs.). The study of Katz *et al*.^31^ does not address any of the crucial issues that should be taken into account before proposing any changes in the subfamilial classification, such as the elucidation of the controversial identity of the type species of *Trichomycterus* (*T. nigricans*), a reasonable sampling of the traditional trichomycterine genera (*Hatcheria*, *Rhizosomichthys*, and *Silvinichthys* are lacking), and the taxonomic allocation of the large number of species that remained in a polyphyletic *Trichomycterus*. The study also ignores most morphological characters proposed in previous studies for delimiting subgroups of their *Cambeva*^74,77,78^. Katz *et al*.^31^ further included in the new genus ten species neither examined for osteology nor sampled for DNA based on “general external appearance and occurrence in the same basins that *Cambeva* is distributed”. The arbitrary creation of new taxon based on an equivocal anatomical character possibly incur in a setback instead of a significant advance in trichomycterid taxonomy.

Finally, the phylogenetic hypothesis proposed here provides a strong framework for future analyses of biogeography, niche modeling, and evolutionary history of multiple traits. At the same time, it also highlights the major challenges to understanding of the systematics of this remarkable family of catfishes.

## Material and Methods

### Taxon sampling

The procedures used for the sampling, maintenance and analysis of the tissue fishes samples are in agreement with Brazilian law regulated by the National Council for the Control of Animal Experimentation (CONCEA) approved by the protocol 1058/2017 and ethical principles in animal research formulated by the Brazilian Society of Science in Laboratory Animals and authorized by the Bioscience Institute/UNESP Ethics Committee on the Use of Animals (CEUA). Tissue samples and voucher specimens used in this study are deposited in the collections of Laboratório de Biologia e Genética de Peixes, Universidade Estadual Paulista, Botucatu, Brazil (LBP), Instituto Nacional de Pesquisa da Amazônia, Manaus, Brazil (INPA), The Academy of Natural Sciences of Drexel University, Philadelphia, USA (ANSP), Colección de Zoologia de la Universidad del Tolima, Ibagué, Colombia (CZUT-IC) and Colección Zoologica de Referencia del Museo de Ciencias Naturales Federico Carlos Lehmann Valencia del INCIVA (IMCN), Cali, Colombia. Supplementary Table 1 synthesizes pertinent data from all samples belonging to the ingroup and outgroups. Our analysis included representatives of the all eight subfamilies, and from 30 genera and 139 species of Trichomycteridae with a total of 153 individuals. Representatives of all remaining families of Loricarioidei were included as outgroups and the resulting trees were rooted in the characiform *Leporinus striatus*.

### Library preparation, target enrichment and sequencing of UCEs

DNA extractions were carried out from approximately 25 mg of tissue using Qiagen DNeasy Tissue kits following the manufacturer’s protocols. After a visual analysis of the quality of DNA on the agarose gel, we quantified 2 µl of each sample using fluorometry (Qubit, Life Technologies) to obtain an ideal concentration between 10–40 ng/µl were used for the analysis. Library preparation and sequencing were performed at Arbor Biosciences (AB; arborbiosci.com; Ann Arbor, MI, USA). AB staff sheared 1–2 µg of DNA to 400–600 bps in length using a Diagenode Bioruptor Standard (UCD 200) with 6–8 cycles of sonication (depending on DNA quality) to prepare the libraries. The DNA libraries from 153 individuals were prepared using the Nextera (Epicentre Biotechnologies, Inc.) library preparation protocol for solution-based target enrichment following Faircloth *et al*.^2^ and increasing the number of PCR cycles following the tagmentation reaction to 20 as recommended by Faircloth *et al*.^5^. AB staff used the Nextera library preparation protocol of *in vitro* transposition followed by PCR to prune the DNA and attach sequencing adapters^87^. The Epicentre Nextera kit was used to prepare transposase-mediated libraries with insert sizes averaging 100 bp (95% CI: 45 bp) following Adey *et al*.^87^. The libraries were enriched using a probe set developed for application to ostariophysan fishes to generate sequences data for approximately 2500 UCE loci^16^. DNA was converted to Illumina sequencing libraries with a slightly modified version of the NEBNext(R) Ultra (TM) DNA Library Prep Kit for Illumina(R). After ligation of sequencing primers, libraries were amplified using KAPA HiFi HotStart ReadyMix (Kapa Biosystems) for six cycles using the manufacturer’s recommended thermal profile and dual P5 and P7 indexed primers see Meyer *et al*.^88^ for primer configuration^89^. After purification with SPRI beads, libraries were quantified with the Quant-iT(TM) Picogreen(R) dsDNA Assay kit (ThermoFisher). AB staff enriched pools comprising 100 ng each of eight libraries (800 ng total) using the MYbaits(R) Target Enrichment system (MYcroarray) followed manual version 3.0. After capture cleanup, the bead-bound library was resuspended in the recommended solution and amplified for 10 cycles, using a universal P5/P7 primer pair and KAPA HiFi reagents. After purification, each captured library pool was quantified with PicoGreen and combined with all other pools in projected equimolar ratios prior to sequencing. Sequencing was performed across two Illumina HiSeq paired-end 100 bp lanes using v4 chemistry.

### Sequence data processing

The standard PHYLUCE pipeline was used for processing target-enriched UCE data^90^. The matrices used in this study were deposited at figshare (doi: 10.6084/m9.figshare.7857485) and sequences are available at NCBI Sequence Read Archive (SRA) submissions: (PRJNA530617). After sequencing, reads were trimmed for adapter contamination, low-quality bases and sequences containing ambiguous bases, using the Illumiprocessor software pipeline, included in the PHYLUCE. We assembled reads and generated consensus contigs for each species using ABySS using a kmer value of 55 (version 2.0.2)^91^.

Following assembly, we screened the resulting assemblies for those contigs matching enriched UCE loci using the “match_contigs_to_probes” program and discarded putative paralogs with the standard PHYLUCE algorithm. We created a fasta file containing all data for all taxa. The monolithic fasta files were used to generate the alignments with MUSCLE^92^ and the resulting alignments were trimmed, using the algorithm implemented by the seqcap_align_2.py script within PHYLUCE. Every alignment was cleaned from the locus name using “phyluce_align_remove_locus_name_from_nexus_lines”. From the trimmed alignments, we created three matrices with 50, 75, and 90% of completeness in order to evaluate the role of missing data in our matrices, tree topology and clade support values. For each matrix we prepared a concatenated alignment in phylip format and every matrix was analyzed using maximum likelihood (ML) algorithm in RAxML v8.2.X^93^ to compare the topologies with different levels of completeness.

### Phylogenetic analyses

The best-fitting partitioning scheme was obtained using the Sliding-Window Site Characteristics (SWSC) approach^94^ to divide each UCE into three data blocks, based on the pattern of variation of entropy, multinomial likelihood and GC content to generate a partition that accounts for heterogeneity in rates and patterns of evolution within each UCE. Posteriorly the data blocks were analyzed using standard algorithms in PartitionFinder v2^95^. The best partitioning scheme grouped loci with having the same substitution model to be used in subsequent analyses. We performed maximum likelihood (ML) inferences on the three concatenated matrices using RAxML ver. 8.1.3^93^, assuming a general time reversible model of rate substitution and gamma-distributed rates among sites (GTRGAMMA). The number of alternative runs and *a posteriori* bootstrapping analysis were conducted using the autoMRE function of bootstopping criteria^96^. Bayesian inference was performed in ExaBayes version 1.5^97^, with two independent runs, each with four chains (one cold and three heated) with 1 million iterations with priors for parameters by default. Tree space was sampled every 100 generations to yield a total of 10,001 trees. We assessed convergence of the posterior distribution examining the ESS > 200 (effective sample size) and evaluating posterior trace distribution in Tracer v1.6.0^98^. We obtained the 50% most credible set of trees from the posterior distribution of possible topologies using the consensus algorithm of ExaBayes with 25% burn-in.

A coalescent analysis of species tree was inferred from individual gene trees to account for heterogeneous gene histories that may influence accurate resolution of phylogenetic relationships^51^. Optimal gene trees for each UCEs under maximum likelihood in RAxML were generated and the unrooted gene trees were used to estimate the true species tree in ASTRAL v 5.6.1.^99^, under the multi-species coalescent model and bootstrap option.

To compare the topologies obtained for each completeness matrix and for each inference method we used the software ViPhy^33^ which incorporate information on similarity scores and allow highlighting of similar structures in multiples trees. We also explored different alternative hypotheses of relationships in Trichomycteridae according with previous morphological and molecular studies. To test the monophyly of Glanapteryginae and Sarcoglanidinae, as well as the different hypotheses of relationships of *Potamoglanis*, we estimated first the best constrained tree for every hypotheses using the concatenated matrix of 75% afterward we compared the best constrained tree with unconstrained tree using Shimodaira-Hasegawa (SH) test^100^ through the -f option in RAxML, and subsequently, p-values were extracted to do Bonferroni correction for the different comparisons using “padjust” in R.

### Ethical approval and informed consent

The procedures used for the sampling, maintenance and analysis of the tissue fishes samples are in agreement with Brazilian law regulated by the National Council for the Control of Animal Experimentation (CONCEA) approved by the protocol 1058/2017 and ethical principles in animal research formulated by the Brazilian Society of Science in Laboratory Animals and authorized by the Bioscience Institute/UNESP Ethics Committee on the Use of Animals (CEUA).

## Supplementary information

Supplementary material.

## Data Availability

The matrices used in this study were deposited at figshare (doi: 10.6084/m9.figshare.7857485) and sequences are available at NCBI Sequence Read Archive (SRA) submissions: (PRJNA530617).
