# Supplementary material for: Phylogenomic analysis of trichomycterid catfishes (Teleostei: Siluriformes) inferred from ultraconserved elements
Source: Sci Rep. 2020 Feb 14;10:2697. doi: 10.1038/s41598-020-59519-w (PMC7021825; doi:10.1038/s41598-020-59519-w)
Supplement: Supplementary file 1 — Supplementary material. [file 41598_2020_59519_MOESM1_ESM.pdf]

Supplementary material

Phylogenomic analysis of trichomycterid catfishes (Teleostei: Siluriformes) inferred from ultraconserved elements

Luz E. Ochoa[a,b], Aléssio Datovo[b], Carlos DoNascimiento[c], Fabio F. Roxo[a], Mark H. Sabaj[d], Jonathan Chang[e], Bruno F. Melo[a], Gabriel S. C. Silva[a], Fausto Foresti[a], Michael Alfaro[e], Claudio Oliveira[a].

[a]*Departamento de Morfologia, Instituto de Biociências, Universidade Estadual Paulista, Botucatu, SP, Brazil*

[b]*Museu de Zoologia da Universidade de São Paulo, São Paulo, SP, Brazil*

[c]*Instituto de Investigación de Recursos Biológicos Alexander von Humboldt, Villa de Leyva, Boyacá, Colombia*

[d]*The Academy of Natural Sciences of Drexel University, Philadelphia, PA, USA*

[e]*Department of Ecology and Evolutionary Biology, University of California, Los Angeles, CA, USA*

Corresponding author

Luz E. Ochoa - luzeocho@gmail.com

Table 1. Taxonomic sampling, catalog and tissue number, geographic distribution of the samples included in this study.

Subfamily	Genus	Species	Specimen Voucher	Tissue Voucher	River	Basin	Country	Acession Number SRA	Coordinates	
Trichogeninae	*Trichogenes*	*Trichogenes longipinnis*	LBP3862	22411	Cachoeira do amor	Atlantico	Brazil	SAMN11320811	S23°21'06.8’	W 44°45'48.9"
Copionodontinae	*Copionodon*	*Copionodon orthiocarinathus*	LBP17354	17354	Mucujê	Paraguaçu	Brazil	SAMN11320812	S 12°57'04"	W 41°16'38"
		Copionodon pecten	LBP17357	38993	Mucujê	Paraguaçu	Brazil	SAMN11320813	S 12°55'32"	W 41°20'04"
		Copionodon sp.	LBP17361	59311	Mucujê	Paraguaçu	Brazil	SAMN11320814	S 13°17'20"	W 41°16'04"
	Glaphyropoma	*Glaphyropoma spinosum*	LBP17359	17359	Gruta dos torres	Paraguaçu	Brazil	SAMN11320815	S 12°52'40"	W 41°18'43"
Glanapteryginae	*Listrura*	*Listrura camposi*	LBP7438	35362	Riacho sem nome	Ribeira do Iguape	Brazil	SAMN11320816	S 24°15'06.3"	W 47°14'53.8"
		Listrura picinguabae	LBP3864	22423	Afluente rio da Fazenda	Ribeira do Iguape	Brazil	SAMN11320817	S 23°20'57.6’	W 44°51'03.4"

Subfamily	Genus	Species	Specimen Voucher	Tissue Voucher	River	Basin	Country	Acession Number SRA	Coordinates	
	Pygidianops	*Pygidianops* sp.1	ANSP190504	2115	Asita	Orinoco	Venezuela	SAMN11320818	N 05°5'20"	W 65°50'32.7"
		Pygidianops sp. 2	ANSP190505	2113	Asita	Orinoco	Venezuela	SAMN11320819	N 05°5'20"	W 65°50'32.7"
	Typhlobelus	*Typhlobelus guacamaya*	ANSP190503	2114	Cuao	Orinoco	Venezuela	SAMN11320820	N 4°59'52.4"	W 67°36'3.6"
	New genus	*Undescribed genus*	LBP16842	69447	Fau	Ribeira do Iguape	Brazil	SAMN11320821	S 24°12'26.5"	W 47°28'36.9"
Sarcoglanidinae	*Microcambeva*	*Microcambeva barbata*	LBP21985	21985	Aldeia Velha	Paraíba do Sul	Brazil	SAMN11320822	S 22°29'17.1"	W 42°16'14.5"
	Sarcoglanis	*Sarcoglanis simplex* 1	ANSP179212	872	Ireng	Takutu	Guyana	SAMN11320823	N 04°1'10"	W 59°36'6"
		Sarcoglanis simplex 2	ANSP180021	2201	Takutu	Takutu	Guyana	SAMN11320824	N 03°21'18"	W 59°49'51"
	Stauroglanis	*Stauroglanis gouldingi*	LBP3159	19301	Negro	Amazonas	Brazil	SAMN11320825	S 02°45'16.6"	W 59°37'30.9"
Tridentinae	*Potamoglanis*	*Potamoglanis hasemani 1*	LBP4483	24450	Negro	Amazonas	Brazil	SAMN11320827	S 00°53'18.6"	W 62°40'36.1"
		Potamoglanis hasemani 2	LBP4483	24450	Negro	Amazonas	Brazil	SAMN11320826	S 00°53'18.6"	W 62°40'36.1"
	Tridens	*Tridens* sp.2 (madeira)	LBP12070	12070	Madeira	Amazonas	Brazil	SAMN11320828	S 8°11'46"	W 63°51'48"
	Tridentopsis	*Tridentopsis pearsoni*	LBP13944	13944	Branco	Amazonas	Brazil	SAMN11320829	S 10°45.710'	W 68°23.564"
	Tridensimilis	*Tridensimilis brevis*	LBP13940	13940	Tapajos	Amazonas	Brazil	SAMN11320830	S 04°55'58.8"	W 56°51'51.6"
Stegophilinae	*Acanthopoma*	*Acanthopoma annectens*	ANSP181146	890	Amazonas	Amazonas	Perú	SAMN11320831	S 03°40'36"	W 73°14'37"
	Haemomaster	*Haemomaster venezuelae*	INPA43789	10704	Xingu	Amazonas	Brazil	SAMN11320832	S 03°33'10.7"	W 51°51'22.2"
	Henonemus	*Henonemus intermedius*	LBP2394	16454	Araguaia	Amazonas	Brazil	SAMN11320833	S 15°53'35.2"	W 52°15'00.9"
		Henonemus punctatus	LBP4146	23679	Jurua	Amazonas	Brazil	SAMN11320834	S 07°26'35.5'	W 73°03'33.5"
		Henonemus sp.	ANSP197334	12772	Jarauçu	Amazonas	Brazil	SAMN11320835	S 01°51'30.4"	W 52°26'46.3"
	Homodiaetus	*Homodiaetus anisitsi*	LBP13194	55163	Camaquã	Lagoa dos Patos	Brazil	SAMN11320836	S 30°44'28.8"	W 51°46'25.1"
		Homodiaetus passarellii	LBP2502	16505	Macacu	Atlantico	Brazil	SAMN11320837	S 22°31'31.6"	W 42°41'17.2"
	Megalocentor	*Megalocentor echthrus*	ANSP199997	4732	Itaya	Amazonas	Perú	SAMN11320838	S 03°43'25"	W 73°12'26"
	Ochmacanthus	*Ochmacanthus alternus*	LBP4351	24088	Igarapé do cajual	Amazonas	Brazil	SAMN11320839	N 03°11'00.8"	W 60°33'20.0"
		Ochmacanthus sp.1 (Itaya)	ANSP191769	4748	Itaya	Amazonas	Brazil	SAMN11320840	S 03°49'59"	W 73°18'07"
		Ochmacanthus sp.2 (Itatá)	ANSP197613	11339	Itaya	Amazonas	Brazil	SAMN11320841	S 03°37'14.6"	W 51°49'15"
		Ochmacanthus reinhardti	LBP10987	50481	Lajeado	Amazonas	Brazil	SAMN11320842	S 10°26'23.5"	W 65°20'34.1"
	Pareiodon	*Pareiodon microps*	ANSP191783	4707	Amazonas	Amazonas	Brazil	SAMN11320843	S 3°43'25"	W 73°12'26"
	Pseudostegophilus	*Pseudostegophilus cf nemurus* (Orinoco)	ANSP198977	13134	Apure	Orinoco	Venezuela	SAMN11320844	N 07°49'2.8"	W 67°21'41.3"
		Pseudostegophilus nemurus	LBP1581	11769	Das Garças	Araguaia	Brazil	SAMN11320845	S 15°54'18.1"	W 52°19'24.2"

Subfamily	Genus	Species	Specimen Voucher	Tissue Voucher	River	Basin	Country	Acession Number SRA	Coordinates	
		Pseudostegophilus paulensis	LBP6738	6738	Tiete	Paraná	Brazil	SAMN11320846	S 21°11'35’	W 49°07'22'
		Pseudostegophilus sp	LBP5133	26235	Paraguai	Paraná	Brazil	SAMN11320847	S 16°06'66"	W 57°44'33"
	Stegophilus	*Stegophilus panzeri*	LBP8619	43462	Tapajos	Amazonas	Brazil	SAMN11320848	S 14°08'39.8'	W 56°05'48.6"
Vandelliinae	*Paracanthopoma*	*Paracanthopoma parva*	LBP2498	15495	Araguaia	Amazonas	Brazil	SAMN11320849	S 15°42'43.4"	W 52°15'32.1"
		Paracanthopoma sp.1 (Nanay)	ANSP199868	4948	Nanay	Amazonas	Perú	SAMN11320850	S 03°45'8.8"	W 73°17'0.1"
		Paracanthopoma sp.2 (Xingu)	ANSP198315	12408	Xingu	Amazonas	Perú	SAMN11320851	S 03°24'54.3"	W 51°42'42.1"
		Paracanthopoma sp. (Jari)	LBP5245	26466	Jari	Amazonas	Brazil	SAMN11320852	S 00°50’38’’	W 52°28’59’’
		Vandellia beccarii	LBP10155	47557	Apure	Orinoco	Venezuela	SAMN11320853	N 07°37'24.4'	W 66°24'48.0"
		Vandellia cirrhosa	ANSP191329	3936	Ventuari	Orinoco	Venezuela	SAMN11320854	N 5°3'2.6"	W 65°37'28.5"
		Vandellia sanguinea	LBP15976	66164	Xingu	Amazonas	Brazil	SAMN11320855	S 13°29'41.8"	W 53°04'57.7"
		Vandellia sp.1 (Araguaia)	LBP1631	11765	Araguaia	Amazonas	Brazil	SAMN11320856	S 15°53'35.2"	W 52°15'00.9"
		Vandellia sp. 2 (Araguaia)	LBP2477	16449	Araguaia	Amazonas	Brazil	SAMN11320857	S 15°53'35.2"	W 52°15'00.9"
		Vandellia sp. 3 (Araguaia)	LBP2477	16450	Araguaia	Amazonas	Brazil	SAMN11320858	S 15°53'35.2"	W 52°15'00.9"
		Vandellia sp. (Perú)	LBP14854	57887	Amazonas	Amazonas	Perú	SAMN11320859		
		Vandellia sp. (Tamshiyacu)	ANSP197186	11246	Tamshiyacu	Amazonas	Perú	SAMN11320860	S 04°1'41.3"	W 73°8'45.2"
		Vandellia sp. (Ventuari)	LBP5342	26973	Ventuari	Orinoco	Venezuela	SAMN11320861	S 00°29’09’’	W 52°41’29’’
Trichomycteridae	*Bullockia*	*Bullockia maldonadoi* 1	LBP3112	3112	La Laja	Bio Bio	Chile	SAMN11320862	S 37°12'54.8"	W 72°26'49.1"
		Bullockia maldonadoi 2	LBP3112	19793	La Laja	Bio Bio	Chile	SAMN11320863	S 37°12'54.8"	W 72°26'49.1"
	Cambeva	*Cambeva balios*	LBP607	7358	Tainhas	Taquari-Antas	Brazil	SAMN11320864	S 29°24,109’	W 50°27,026
		Cambeva cubataonis	LBP3123	19690	Itapocu	Atlantico	Brazil	SAMN11320865	S 26°28'17.2"	W 49°10'55.1"
		Cambeva davisi (Iguaçu)	LBP1222	10598	Iguaçu	Atlantico	Brazil	SAMN11320868	S 25°52.438’	W 49°43.404'
		Cambeva davisi	LBP6383	29744	Preto	La Plata	Brazil	SAMN11320867	S 23°57.270'	W 51°06.843'
		Cambeva iheringi	LBP4512	24563	Paranapiacaba	Paraná	Brazil	SAMN11320866	S 23°46'13.2"	W 46°18'39.6"
		Cambeva perkos	LBP17033	66403	Uruguay	Uruguay	Brazil	SAMN11320870	S 28°08'03.4"	W 52°18'40.7"
		Cambeva poikilos	LBP14693	61154	Carreiro	Uruguay	Brazil	SAMN11320871	S 28°43'54.0"	W 51°50'23.6"
		Cambeva pascuali	LBP23323		Tamandua	Paranapanema	Brazil	SAMN11320869	S 23°13'27.06"	W 48ª31'45.34"
		Cambeva sp *(Santa Catarina)*	LBP3121	19686	Itapocu	Atlantico	Brazil	SAMN11320872	S 26°28'17.2"	W 49°10'55.1"
		Cambeva stawiarski	LBP16165	66905	Cachoeira	Paraguay	Brazil	SAMN11320873	S 25°22'09.9"	W 54°06'49.7"

Subfamily	Genus	Species	Specimen Voucher	Tissue Voucher	River	Basin	Country	Acession Number SRA	Coordinates	
		Cambeva zonatus	LBP2653	17409	Ribeira do Iguape	Ribeira do Iguape	Brazil	SAMN11320874	S 24°41'11.8"	W 48°59'40.3"
	Eremophilus	*Eremophilus mutisii*	no voucher	11306	Laguna de Funeque	Magdalena	Colombia	SAMN11320875	N 05°27'15"	W 73°44'40"
	Ituglanis	*Ituglanis amazonicus*	LBP2442	16211	Araguaia	Amazonas	Brazil	SAMN11320876	S 15°52'40.4’’	W 52°18'15.5’’
		Ituglanis boitata	LBP14546	60865	Jacui	Lagoa dos Patos	Brazil	SAMN11320877	S 29°33'53.4"	W 53°17'08.1"
		Ituglanis cf *amazonicus*	LBP11003	50532	Madeira	Amazonas	Brazil	SAMN11320878	S 10°26'23.5"	W 65°20'34.1"
		Ituglanis cf *eichorniarum*	LBP10777	49859	Paraguay	Paraguay	Brazil	SAMN11320879	S 18°25'24.4"	W 54°50'05.9"
		Ituglanis cf *goya*	LBP17131	68592	Das Almas	Tocantins	Brazil	SAMN11320880	S 13°45'18.5"	W 47°27'20.0"
		Ituglanis cf *parkoi*	LBP7995	37376	Tapajos	Amazonas	Brazil	SAMN11320881	S 14°08'14.1"	W 56°05'48.6"
		Ituglanis eichorniarum	LBP4686	24825	Paraguay	Paraguay	Brazil	SAMN11320882	S 14°41'44’	W 57°15'35'
		Ituglanis goya	LBP17137	68599	Dos Couros	Tocantins	Brazil	SAMN11320883	S 14°09'46.8"	W 47°37'52.4"
		Ituglanis herberti	LBP676	8028	Pirai	Paraná	Brazil	SAMN11320884	S 16°25,680'	W 56°25,143'
		Ituglanis parahybae	LBP10730	49719	Macabu	Paraíba do Sul	Brazil	SAMN11320885	S 22°04'07.8"	W41°54'36.2"
		Ituglanis parkoi	LBP14153	59188	Tapajos	Amazonas	Brazil	SAMN11320886	S 04°43'13.8"	W 56°44'24.3"
		Ituglanis ramiroi	LBP15293	63261	Rio São Bernardo	Tocantins	Brazil	SAMN11320887	S 13°44'15.9"	W 46°21'48.8"
		*Ituglanis*_sp. (Araguaia)	LBP1857	13264	Insula	Araguaia	Brazil	SAMN11320888	S 15°32'54.2"	W 52°12'17.7"
		Ituglanis sp. (Das Brancas)	LBP19465	77969	Das Brancas	Tocantins	Brazil	SAMN11320889	S 14º53'50.7"	W 47º35'00.2"
		Ituglanis sp. (Cuiaba)	LBP12960	55688	Rio Cuiaba	Paraguai	Brazil	SAMN11320890	S 17°49'51.6"	W 57°23'42.8"
		Ituglanis sp. (Joao dias)	LBP7667	36475	Corrego João Dias	La Plata	Brazil	SAMN11320891	S 20°20'33.6'	W 55°43'34.2"
		Ituglanis sp. (Ribeira)	LBP7416	35678	Batatau	Ribeira do Iguape	Brazil	SAMN11320892	S 24°35'25.5"	W 48°16'29.9"
		Ituglanis sp. (Tapajos)	LBP16129	66849	Tapajos	Amazonas	Brazil	SAMN11320893	S 04°33'09.7"	W 56°17'59.6"
		Ituglanis sp. (Xingu)	INPA47781	11584	Xingu	Amazonas	Brazil	SAMN11320894	S 03°37'14.6"	W 51°49'15"
	Scleronema	*Scleronema minutum*	LBP3310	19841	Arroio dos Corrientes	Atlantico	Brazil	SAMN11320895	S 31°28'46.3"	W 52°12'46.9"
	Trichomycterus	*Trichomycterus aff spilosoma*	LBP19339	77975	Dos Bocas	Atlantico	Ecuador	SAMN11320896	S03°16'07.6"	W079°44'14,8''
		Trichomycterus aff striatus	LBP19846	77969	Magdalena	Magdalena	Colombia	SAMN11320897	N 04°29'11.0’’	W 75°15'10"
		Trichomycterus albinotatus	LBP6326	29802	Paraiba do sul	Paraíba do Sul	Brazil	SAMN11320898	S 22°39'19.6"	W 44°34"44.0"
		Trichomycterus alternatus	LBP1014	10261	Chopoto	São francisco	Brazil	SAMN11320899	S 21°08.947'	W 43°23.973'
		Trichomycterus areolatus	LBP3118	19819	Maichin	Maichin	Chile	SAMN11320900	S 39°20'41.3"	W 71°34'27.0"
		Trichomycterus banneaui	LBP19847	77973	Magdalena	Magdalena	Colombia	SAMN11320901	N 03º43'3,971"	W 74º55'43.958"

Subfamily	Genus	Species	Specimen Voucher	Tissue Voucher	River	Basin	Country	Acession Number SRA	Coordinates	
		Trichomycterus brasiliensis	LBP10675	49540	São Francisco	São francisco	Brazil	SAMN11320902	S 18°13'48.0"	W 45°13'52.7"
		Trichomycterus cachiraensis	LBP19832	77957	Galvanes	Magdalena	Colombia	SAMN11320903	N 07°42'21.3"	W 73°02'09.2"
		Trichomycterus candidus	LBP11630	58011	Paraná	Paraná	Brazil	SAMN11320904	S 19°41'32.0"	W 47°42'58.6"
		Trichomycterus chapmani 1	CZUT-IC17749	Q53	Santo Domingo	Magdalena	Colombia	SAMN11320905	N 04º 31' 19,4"	75º 37' 33,7"
		Trichomycterus chapmani 2	CZUT-IC17061	L9	La vieja	Cauca	Colombia	SAMN11320906	N 12º 42' 47,73"	W 83º 51' 6,34"
		Trichomycterus chiltoni	ANSP180474	940	Andalien	Andalien	Chile	SAMN11320907	S 36°48'30"	W 73°1'32"
		Trichomycterus cf. *areolatus* (La Laja)	LBP3113	3113	La Laja	Bio Bio	Chile	SAMN11320908	S 37°12'54.8"	W 72°26'49.1"
		Trichomycterus cf. *areolatus* (Maichin)	LBP3118	19820	Maichin	Maichin	Chile	SAMN11320909	S 39°20'41.3"	W 71°34'27.0"
		Trichomycterus cf. *auroguttatus*	LBP8374	40446	Paraiba do sul	Paraíba do Sul	Brazil	SAMN11320910	S 21°14'07.4'	W 43°30'50.5"
		Trichomycterus cf. *banneaui*	LBP19537	77971	Magdalena	Magdalena	Colombia	SAMN11320911	N 06°02'09.0"	W 74°58'56.0"
		Trichomycterus cf. *brasiliensis* 1	LBP8060	37829	Grande	La Plata	Brazil	SAMN11320912	S 22°40'33.2"	W 45°40'58.3"
		Trichomycterus cf. *brasiliensis* 2	LBP6247	29199	Grande	La Plata	Brazil	SAMN11320913	S 21°19'44.8"	W 46°30'04.6"
		Trichomycterus cf. *brasiliensis* 3	LBP10276	47976	Grande	La Plata	Brazil	SAMN11320914	S 20°17'11.6'	W 46°34'55.0"
		Trichomycterus cf. *guianensis*	ANSP179111	918	Orokang	Mazaruni	Guyana	SAMN11320915	N 5°31'31"	W 60°13'56"
		Trichomycterus cf. *knerii*	LBP18717	18717	Meta/caño Guamal	Orinoco	Colombia	SAMN11320916	N 03°52'10.413"	W 73°46'15.138"
		Trichomycterus cf. *oroyae*	LBP3255	3255	Chontabamba	Amazonas	Perú	SAMN11320917	S 10º36'06,6''	W 075º29'10,8''
		Trichomycterus cf. *septemradiatus*	LBP5939	28065	São Domingos	Grande	Brazil	SAMN11320918	S 21°22'48.1"	W 46°28'29.7"
		Trichomycterus cf *taenia*	CZUT-IC NJ82 T24437		Dagua	Pacifico	Colombia	SAMN11320919	N 03°37'14.104"	W 76°40'53.407"
		Trichomycterus guianensis	LBP17444	69015	Potaro	Essequibo	Guyana	SAMN11320920	N 05°20'16.0''	W 59°33'57.0''
		Trichomycterus immaculatus	LBP8351	40419	Piçarrão	Doce	Brazil	SAMN11320921	S 19°40'53.8'	W 43°00'50.1"
		Trichomycterus itatiaye	LBP16356	62282	Paraiba do sul	Paraíba do Sul	Brazil	SAMN11320922	S 22°28'46.595'	W 44°34'1.978"
		Trichomycterus mimosensis	LBP8290	38358	Jequitinhonha	Jequitinhonha	Brazil	SAMN11320923	S 17°13'14.0'	W 42°35'46.0"
		Trichomycterus nigroauratus	LBP6301	29341	Itagaçaba	Paraíba do Sul	Brazil	SAMN11320924	S 22°39'26.3"	W 44°45'49.8"
		Trichomycterus pauciradiatus	LBP16323	61977	São Francisco	São francisco	Brazil	SAMN11320925	S 20°57'20.1"	W 43°45'43.9"
		Trichomycterus pirabitira	LBP18381	72641	Grande	La Plata	Brazil	SAMN11320926	S 21°8′53″	W 46°14′95″
		Trichomycterus piratymbara	LBP9004	42138	Grande	La Plata	Brazil	SAMN11320927	S 20°37'25.4"	W 46°13'49.5"
		Trichomycterus pradensis	LBP8291	38359	Jequitinhonha	Jequitinhonha	Brazil	SAMN11320928	S 17°13'14.0'	W 42°35'46.0"
		Trichomycterus punctulatus	ANSP180733	905	Pisco	Pisco	Perú	SAMN11320929	S 13°40'	W 75°46'18"

Subfamily	Genus	Species	Specimen Voucher	Tissue Voucher	River	Basin	Country	Acession Number SRA	Coordinates	
		Trichomycterus quechuorum	ANSP180572	912	Mapacho	Mapacho	Perú	SAMN11320930	S 13°37'3"	W 71°24'4"
		Trichomycterus reinhardti	LBP16302	61942	São Francisco	São francisco	Brazil	SAMN11320931	S 20°11'40.5"	W 44°06'05.1"
		Trichomycterus ruitoquensis	LBP19838	77956	Magdalena	Magdalena	Colombia	SAMN11320932	N 07°04'08.5"	W 73°04'13.5"
		Trichomycterus sandovali	LBP19833	77946	Don Juan cave	Magdalena	Colombia	SAMN11320933	N 06°48'13.5"	W 73°16'23.1"
		Trichomycterus septemradiatus	LBP6550	31670	Das Velhas	São francisco	Brazil	SAMN11320934	S 20°00'37.1"	W 43°58'08.3"
		Trichomycterus sp. (Arica-Mirim)	LBP7640	36427	Afluente rio Aricá-Mirim	La Plata	Brazil	SAMN11320935	S 15°46'03.8"	W 55°30'44.5"
		Trichomycterus sp. (Bonito)	LBP11648	58088	Bonito	Paraná	Brazil	SAMN11320936	S 18°37'15.6"	W 48°36'30.7"
		Trichomycterus sp. (Casca)	LBP7669	36484	Afluente rio da Casca	Paraguai	Brazil	SAMN11320937	S 16°55.491'	W 55°45.191'
		Trichomycterus sp. (Corrego)	LBP10236	47789	Corrego sem nome	La Plata	Brazil	SAMN11320938	S 15°46'36.9'	W 55°30'09.8"
		Trichomycterus sp. (Coxipo-açu)	LBP7674	36488	Afluente rio Coxipo-Açu	La Plata	Brazil	SAMN11320939	S 15°07.362'	W 55°58.563'
		Trichomycterus sp. (Grande)	LBP10282	47992	Araguari	La Plata	Brazil	SAMN11320940	S 20°08'47.3'	W 46°40'11.1"
		Trichomycterus sp. (Grotão)	LBP11631	58082	Paraná	Paraná	Brazil	SAMN11320941	S 19°41'32.0"	W 47°42'58.6"
		Trichomycterus sp (Manco)	LBP19834	77958	Manco	Magdalena	Colombia	SAMN11320942	N 06°50'58.1"	W 72°59'19.2"
		Trichomycterus sp (Paria)	ANSP191470	2083	Peninsula de Paria	Atlantico	Venezuela	SAMN11320943	N 10°39'	W 62°34'
		Trichomycterus sp. (Turvo)	LBP9001	42123	Grande	La Plata	Brazil	SAMN11320944	S 20°35'44.9"	W 46°13'16.6"
		Trichomycterus sp. (São Francisco)	LBP11842	58147	Córrego da Agua Santa	São francisco	Brazil	SAMN11320945	S 20°08'47.3"	W 46°40'11.1"
		Trichomycterus sp. (Sepotuba)	LBP8563	43332	Sepotuba	Paraguay	Brazil	SAMN11320946	S 14°20'32.6"	W 57°31'22.5"
		Trichomycterus sp. (Samaná Norte)	CZUT-IC17547	8	Samaná Norte	Magdalena	Colombia	SAMN11320947	N 6º11'48.869"	W 74º47'41.107"
		Trichomycterus sp.4 (Riecito)	CZUT-IC20105	562	Riecito	Magdalena	Colombia	SAMN11320948	N 2º0'34.381"	W75º46'41.362"
		Trichomycterus sp. (Rancheria)	IMCN8442	1	Rancheria	Rancheria	Colombia	SAMN11320949		
		Trichomycterus trasandianus 1	LBP19845	77964	Magdalena	Magdalena	Colombia	SAMN11320950	N 4º27'55.7"	W 75º12'21.1"
		Trichomycterus trasandianus 2	CZUT-IC18148	657	Combeima	Magdalena	Colombia	SAMN11320951	N 4º31'02.3"	W 75º18'28.0"
Nematogenyidae	*Nematogenys*	*Nematogenys inermis*	LBP3105		Andalien	Pacifico	Chile	SAMN11320952	S 36°50.304'	W 72°55.642'
Callichthyidae	*Corydoras*	*Callichthys callichthys*	LBP17423	69177	São Francisco	São francisco	Brazil	SAMN11320964	S 19°37'56.4"	W 44°02'47.4"
		Corydoras elegans	LBP14804	57701	Aquarium	Amazonas	Perú	SAMN11320953		
		Corydoras gossei	LBP544	7171	Aquarium	Amazonas	Brazil	SAMN11320954		
	Hoplosternum	*Hoplosternum littorale*	LBP466	7282	Paranapanema	Paranapanema	Brazil	SAMN11320955	S 19°34,630'	W 57°01,123'
Scoloplacidae	*Scoloplax*	*Scoloplax dicra*	LBP11001	50522	Lajeado	Amazonas	Brazil	SAMN11320956	S 10°26'23.5"	W 65°20'34.1"

Subfamily	Genus	Species	Specimen Voucher	Tissue Voucher	River	Basin	Country	Acession Number SRA	Coordinates	
		Scoloplax distolothrix	LBP1938	14340	Itiquira	Piquiri	Brazil	SAMN11320957	S 17°28'13"	W 55°14'46.7"
Loricariidae	*Farlowella*	*Farlowella oxyrryncha*	LBP1558	11509	Das mortes	Amazonas	Brazil	SAMN11320958	S 15°29'57.3"	W 52°12'10.4"
	Lamontichthys	*Lamontichthys filamentosus*	LBP162	4093	Branco	Amazonas	Brazil	SAMN11320959	S 10°03,320'	W 67°51,450'
	Lasiancistrus	*Lasiancistrus saetiger*	LBP9156	42517	Guamá	Amazonas	Brazil	SAMN11320960	S 01°34'28.3"	W 47°02'03.5"
Astroblepidae	*Astroblepus*	*Astroblepus grixalvii*	ANSP188920	188920	Dormilon	Magdalena	Colombia	SAMN11320961	N 06°2'47.5"	W 75°29'24.8"
		Astroblepus sp	LBP19836	77952	Frio	Magdalena	Colombia	SAMN11320962	N 07°04'08.5"	W 73°04'13.5"
Characidae	*Leporinus*	*Leporinus striatus*	LBP3180	16871	Paranapanema	Paraná	Brazil	SAMN11320963	S 23°20'	W 48°34'

Table 2. Summary values describing the number of trimmed reads, number of contigs assembled, total base pair of contigs, number of UCE contigs, mean length of all contigs and their average coverage for samples.

Subfamily	Genus	Species	Voucher	Tissue number	Number of trimmed reads	Contigs assembled	Total bp contigs	UCE contigs	Mean length	Total bp UCES
Trichogeninae	*Trichogenes*	*Trichogenes longipinnis*	LBP3862	22411	2,575,084	13076	7957424	1,713	324	554,165
Copionodontinae	*Copionodon*	*Copionodon orthiocarinathus*	LBP17354	17354	2,649,930	12142	5260402	1,652	759	1,253,244
		Copionodon pecten	LBP17357	38993	2,000,677	8543	3738395	1,486	711	1,056,864
		Copionodon sp.	LBP17361	59311	2,612,582	23120	9688341	1,673	274	458,776
	Glaphyropoma	*Glaphyropoma spinosum*	LBP17359	17359	2,554,783	10115	4218198	1,669	658	1,098,364
Glanapteryginae	*Listrura*	*Listrura camposi*	LBP7438	35362	1,229,746	4325	2721299	1,140	230	262,012
		Listrura picinguabae	LBP3864	22423	3,637,164	4162	2484286	1,118	680	760,094
	Pygidianops	*Pygidianops* sp.1	ANSP190504	2113	5,484,465	36085	15192123	1,349	357	481,481
		Pygidianops sp.2	ANSP190505	2115	4,191,971	33264	14520463	1,317	971	1,278,855
	Typhlobelus	*Typhlobelus guacamaya*	ANSP190503	2114	2,060,441	10035	5709065	1,407	900	1,265,817
	New genus	*Undescribed genus*	LBP16842	69447	1,532,438	7655	4261083	1,389	789	1,095,306
Sarcoglanidinae	*Microcambeva*	*Microcambeva barbata*	LBP21985	21985	335,526	1524	591364	825	321	264,617
	Sarcoglanis	*Sarcoglanis simplex* 1	ANSP179212	872	1,965,651	16050	7708086	1,396	848	1,183,777
		Sarcoglanis simplex 2	ANSP180021	2201	6,483,210	29490	12177368	1,400	859	1,202,544
	Stauroglanis	*Stauroglanis gouldingi*	LBP3159	19301	3,892,538	12136	6098347	1,359	333	451,943
Tridentinae	*Potamoglanis*	*Potamoglanis hasemani* 1	LBP4483	24450	1,205,504	6025	3130825	1,002	209	209,577
		Potamoglanis hasemani 2	LBP4483	23971	1,438,585	10781	5483335	1,326	809	1,073,929
	Tridens	*Tridens* sp. 2 (Madeira)	LBP12070	12070	1,809,411	6719	3671354	1,087	244	264,959
	Tridentopsis	*Tridentopsis pearsoni*	LBP13944	13944	2,120,8	6391	3384015	1,229	777	955,174
	Tridensimilis	*Tridensimilis brevis*	LBP13940	13940	1,340,964	5266	2858644	995	205	204,215
Stegophilinae	*Acanthopoma*	*Acanthopoma annectens*	ANSP181146	890	2,038,992	4948	2938044	1,146	780	893,573
	Haemomaster	*Haemomaster venezuelae*	INPA43789	10704	568,892	1842	832244	776	391	303,698
	Henonemus	*Henonemus intermedius*	LBP2394	16454	2,716,966	7615	4315084	1,314	886	1,164,658
		Henonemus punctatus	LBP4146	23679	1,796,713	3711	2112946	1,100	689	757,526

Subfamily	Genus	Species	Voucher	Tissue number	Number of trimmed reads	Contigs assembled	Total bp contigs	UCE contigs	Mean length	Total bp UCES
		Henonemus sp.	ANSP197334	12772	1,277,443	4624	2665717	1,215	782	950,038
	Homodiaetus	*Homodiaetus anisitsi*	LBP13194	55163	1,473,577	4332	2894165	1,017	708	720,310
		Homodiaetus passarellii	LBP2502	16505	4,460,737	8092	4532053	1,197	722	864,681
	Megalocentor	*Megalocentor echthrus*	ANSP199997	4732	1,083,727	3432	2115847	1,042	686	714,668
	Ochmacanthus	*Ochmacanthus alternus*	LBP4351	24088	477,062	3988	2118212	1,146	708	811,688
		Ochmacanthus sp.1 (Itaya)	ANSP191769	4748	631,989	4568	2494743	1,151	747	859,683
		Ochmacanthus sp.2 (Itatá)	ANSP197613	11339	848,287	5107	2743488	1,167	776	905,513
		Ochmacanthus reinhardti	LBP10987	50481	3,092,031	6605	3326668	1,111	644	715,516
	Pareiodon	*Pareiodon microps*	ANSP191783	4707	2,544,499	7770	3679016	1,206	733	884,259
	Pseudostegophil us	*Pseudostegophilus* cf. *nemurus* (Orinoco)	ANSP198977	13134	2,807,759	8109	3859728	1,219	745	907,922
		Pseudostegophilus nemurus	LBP1581	11769	2,365,757	4401	2545956	1,172	706	827,767
		Pseudostegophilus paulensis	LBP6738	6738	1,214,052	7046	4235997	632	621	392,332
		Pseudostegophilus sp	LBP5133	26235	1,389,668	5440	3226705	1,238	765	947,393
	Stegophilus	*Stegophilus panzeri*	LBP8619	43462	148,566	1621	873199	874	548	478,939
Vandelliinae	*Paracanthopoma*	*Paracanthopoma parva*	LBP2498	15495	1,667,541	4246	2457937	1,056	761	804,078
		Paracanthopoma sp 1 (Nanay)	ANSP199868	4948	1,651,943	6372	3443818	1,058	786	831,110
		Paracanthopoma sp 2 (Xingu)	ANSP198315	12408	1,278,393	4019	2033520	862	601	518,277
		Paracanthopoma sp. (Jari)	LBP5245	26466	2,418,434	9260	4492239	1,008	499	502,673
	Vandellia	*Vandellia beccarii*	LBP10155	47557	4,203,329	17319	6977762	1,007	855	860,930
		Vandellia cirrhosa	ANSP191329	3936	451,062	2420	1636640	840	853	716,888
		Vandellia sanguinea	LBP15976	66164	2,030,908	4431	2611384	981	708	694,309
		Vandellia sp.1 (Araguaia)	LBP1631	11765	1,799,338	5008	2772867	906	745	675,354
		Vandellia sp.2 (Araguaia)	LBP2477	16449	2,509,744	7479	3899098	928	711	659,797
		Vandellia sp.3 (Araguaia)	LBP2477	16450	3,582,938	12596	5934891	957	684	654,135
		Vandellia sp. (Perú)	LBP14854	57887	1,187,003	3262	1916526	895	735	658,087
		Vandellia sp. (Tamshiyacu)	ANSP197186	11246	1,131,701	4328	2996114	977	866	845,847

Subfamily	Genus	Species	Voucher	Tissue number	Number of trimmed reads	Contigs assembled	Total bp contigs	UCE contigs	Mean length	Total bp UCES
		Vandellia sp. (Ventuari)	LBP5342	26973	2,201,698	6842	3438183	931	784	729,985
Trichomycteridae	*Bullockia*	*Bullockia maldonadoi* 1	LBP3112	3112	69,683	664	219602	559	224	125,236
		Bullockia maldonadoi 2	LBP3112	19793	2,763,220	8305	4476703	1,430	786	1,123,436
	Cambeva	*Cambeva balios*	LBP607	7358	2,873,117	16531	7674658	658	468	307,696
		Cambeva cubataonis	LBP3123	19690	2,040,601	8069	4605261	1,305	255	332,293
		Cambeva iheringi	LBP4512	24563	2,058,795	11012	5711264	1,573	849	1,336,039
		Cambeva davisi	LBP6383	29744	1,232,883	8218	4320816	1,541	810	1,247,508
		Cambeva davisi (Iguaçu)	LBP1222	10598	2,095,335	8042	4465295	1,334	241	321,817
		Cambeva pascuali	LBP23323		1,653,916	7121	3874302	1,286	811	1,043,066
		Cambeva perkos	LBP17033	66403	2,606,839	8378	4666732	1,493	841	1,256,105
		Cambeva poikilos	LBP14693	61154	2,366,092	7599	4073261	1,496	758	1,134,105
		Cambeva sp *(Santa Catarina)*	LBP3121	19686	1,839,591	9945	5412209	1,535	862	1,322,549
		Cambeva stawiarski	LBP16165	66905	2,162,482	8092	4407820	1,352	239	323,317
		Cambeva zonatus	LBP2653	17409	1,483,970	7041	4017012	1,416	862	1,220,827
	Eremophilus	*Eremophilus mutisii*	ANSP11306	no voucher	1,406,788	5411	2678729	1,268	661	838,429
	Ituglanis	*Ituglanis amazonicus*	LBP2442	16211	3,185,776	8636	4868257	1,465	275	402,195
		Ituglanis amphipotamus	LBP7416	35678	3,084,996	13996	6561776	1,489	770	1,146,164
		Ituglanis boitata	LBP14546	60865	928,397	5221	2853020	1,409	731	1,029,446
		Ituglanis cf *amazonicus*	LBP11003	50532	860,615	7247	4010231	1,424	806	1,147,357
		Ituglanis cf *eichorniarum*	LBP10777	49859	1,340,525	6170	3545189	1,291	244	315,198
		Ituglanis cf *goya*	LBP17131	68592	1,530,163	6267	3548075	1,362	230	313,575
		Ituglanis cf *parkoi*	LBP7995	37376	1,597,887	8787	4728872	1,469	280	411,147
		Ituglanis eichorniarum	LBP4686	24825	1,023,343	7290	3934977	1,350	271	366,379
		Ituglanis goya	LBP17137	68599	1,139,418	5486	3140687	1,226	226	277,211
		Ituglanis herberti	LBP676	8028	1,848,568	9627	5207612	1,453	287	416,383
		Ituglanis parahybae	LBP10730	49719	525,319	5445	2811435	1,387	695	964,347
		Ituglanis parkoi	LBP14153	59188	4,056,525	10038	5733370	1,517	290	440,251

Subfamily	Genus	Species	Voucher	Tissue number	Number of trimmed reads	Contigs assembled	Total bp contigs	UCE contigs	Mean length	Total bp UCES
		Ituglanis ramiroi	LBP	63261	1,275,546	6121	3474181	1,384	857	1,185,591
		*Ituglanis*_sp (Araguaia)	LBP1857	13283	1,293,835	6321	3659124	1,420	865	1,228,122
		Ituglanis sp (DasBrancas)	LBP19465	77969	1,885,919	7326	3936340	1,406	232	325,727
		Ituglanis sp (Cuiaba)	LBP12960	55688	1,089,983	6115	3407999	1,363	780	1,062,781
		Ituglanis sp (Joao dias)	LBP7667	36475	1,515,489	7062	4073949	1,250	279	348,978
		Ituglanis sp (Tapajos)	LBP16129	66849	1,465,298	5994	3385205	1,317	237	312,493
		Ituglanis sp (Xingu)	INPA11584	11584	1,766,824	7833	3570426	1,221	653	797,903
	Scleronema	*Scleronema minutum*	LBP3310	19841	1,197,170	6972	3627542	1,440	782	1,126,696
	Trichomycterus	*Trichomycterus* aff. *spilosoma*	LBP19339	77975	3,235,129	9625	5359917	1,541	259	398,748
		Trichomycterus aff. *striatus*	LBP19846	77968	4,995,362	7915	4517538	1,360	252	343,370
		Trichomycterus albinotatus	LBP6326	29802	674,307	4740	2443676	1,331	682	907,221
		Trichomycterus alternatus	LBP1014	10261	1,136,511	6372	3342485	1,338	779	1,042,557
		Trichomycterus areolatus	LBP3118	19819	2,820,795	8906	4643046	1,390	235	326,419
		Trichomycterus banneaui	LBP19847	77973	1,327,537	6842	3860594	1,502	802	1,204,079
		Trichomycterus brasiliensis	LBP10675	49540	822,056	4915	2210634	1,371	589	807,201
		Trichomycterus cachiraensis	LBP19832	77957	3,218,412	10335	5654368	1,470	276	405,266
		Trichomycterus candidus	LBP11630	58011	819,318	6328	3256499	1,434	741	1,062,450
		Trichomycterus chapmani 1	CZUT-IC17749	L8	2,950,612	13053	6870330	1,571	342	537,762
		Trichomycterus chapmani 2	CZUT-IC17061	L9	2,724,440	12011	6179518	1,330	302	402,307
		Trichomycterus chiltoni	ANSP180474	940	678,040	5299	2803034	1,319	729	961,950
		Trichomycterus cf. *areolatus* (La Laja)	LBP3113	3113	2,756,814	7878	3868680	1,315	664	872,819
		Trichomycterus cf. *areolatus* (Maichin)	LBP3118	19820	1,088,389	8050	4086049	1,488	772	1,149,312
		Trichomycterus cf. *auroguttatus*	LBP8374	40446	3,161,125	9508	5001009	1,415	259	366,033
		Trichomycterus cf. *banneaui*	LBP19537	77971	2,488,653	12633	6157627	1,507	824	1,241,775
		Trichomycterus cf. *brasiliensis 1*	LBP8060	37829	1,479,875	8382	4425849	1,446	802	1,159,552

Subfamily	Genus	Species	Voucher	Tissue number	Number of trimmed reads	Contigs assembled	Total bp contigs	UCE contigs	Mean length	Total bp UCES
		Trichomycterus cf. *brasiliensis2*	LBP6247	29199	1,390,876	9465	4880824	1,575	819	1,289,937
		Trichomycterus cf. *brasiliensis 3*	LBP10276	47976	1,646,306	9194	4694051	1,579	774	1,222,293
		Trichomycterus cf. *guianensis*	ANSP179111	918	2,009,103	11582	5558685	973	613	596,228
		Trichomycterus cf. *knerii*	LBP18717	18717	3,253,475	9873	5328560	1,504	258	387,392
		Trichomycterus cf. *oroyae*	LBP3255	3255	2,991,667	8144	4005346	1,345	680	914,842
		Trichomycterus cf. *septemradiatus*	LBP5939	28065	3,687,832	10306	5030111	1,524	828	1,261,658
		Trichomcyterus cf *taenia*	CZUT-IC NJ82 T24437	90790	2,159,029	10053	5699383	1,535	330	507,156
		Trichomycterus guianensis	LBP17444	69015	1,301,884	8759	4686001	1,494	854	1,276,601
		Trichomycterus immaculatus	LBP8351	40419	2,062,904	8532	4385595	1,323	256	339,315
		Trichomycterus itatiaye	LBP16356	62282	2,610,414	13671	6052765	1,561	777	1,213,328
		Trichomycterus mimosensis	LBP8290	38358	1,484,105	7567	3811910	1,455	750	1,090,882
		Trichomycterus nigroauratus	LBP6301	29341	2,058,398	13082	5883948	1,503	763	1,146,749
		Trichomycterus pauciradiatus	LBP16323	61977	2,328,959	10974	5238399	1,468	811	1,190,568
		Trichomycterus pirabitira	LBP18381	72641	4,076,534	16242	7638686	1,634	812	1,326,841
		Trichomycterus piratymbara	LBP9004	42138	2,566,246	12432	5681233	1,575	789	1,242,110
		Trichomycterus pradensis	LBP8291	38359	2,599,193	9641	4880259	1,395	245	341,564
		Trichomycterus punctulatus	ANSP180733	905	4,039,139	14931	6588368	1,566	729	1,141,332
		Trichomycterus quechuorum	ANSP180572	912	2,686,894	12970	6456491	1,565	844	1,321,563
		Trichomycterus reinhardti	LBP16302	61942	4,662,949	18616	8714563	1,609	872	1,403,785
		Trichomycterus ruitoquensis	LBP19838	77956	2,499,669	8962	5043610	1,474	257	378,738
		Trichomycterus sandovali	LBP19833	77946	2,924,535	9631	5071729	1,467	262	384,088
		Trichomycterus septemradiatus	LBP6550	31670	2,230,258	15853	7314932	1,625	840	1,365,410
		Trichomycterus sp. (Arica-Mirim)	LBP7640	36427	1,756,399	7251	4448990	1,297	259	335,719
		Trichomycterus sp. (Bonito)	LBP11648	58088	40,059	176	66129	238	164	39,145
		Trichomycterus sp. (Casca)	LBP7669	36484	1,610,059	6953	4322946	1,220	254	309,670

Subfamily	Genus	Species	Voucher	Tissue number	Number of trimmed reads	Contigs assembled	Total bp contigs	UCE contigs	Mean length	Total bp UCES
		Trichomycterus sp. (Corrego)	LBP10236	47789	1,820,364	7232	4423305	1,286	254	326,789
		Trichomycterus sp. (Coxipo-açu)	LBP7674	36488	1,371,402	6382	4001256	1,169	246	287,690
		Trichomycterus sp. (Grande)	LBP10282	47992	2,087,560	10779	5302037	1,495	835	1,248,650
		Trichomycterus sp. (Grotão)	LBP11631	58082	652,418	4396	2101157	1,293	602	778,015
		Trichomycterus sp (Manco)	LBP19834	77958	2,261,658	8597	4848543	1,436	252	362,559
		Trichomycterus sp (Paria)	ANSP191472		2,128,871	11768	6178939	1,575	310	488,863
		Trichomycterus sp. (Turvo)	LBP9001	42123	1,112,897	4427	2187647	1,331	630	839,173
		Trichomycterus sp. (São Francisco)	LBP11842	58147	3,414,540	14634	7246441	1,583	876	1,386,945
		Trichomycterus sp. (Sepotuba)	LBP8563	43332	1,888,949	7040	4167351	1,393	234	325,470
		Trichomycterus sp. (Samaná Norte)	CZUT-IC17547	17547	2,485,700	11084	5049425	1,537	646	992,273
		Trichomycterus sp.4 (Riecito)	CZUT-IC20105	20105	2,527,958	11673	6000276	1,535	294	451,516
		Trichomycterus sp. (Rancheria)	IMCN	91504	2,690,671	11965	5998933	1,620	322	522,117
		Trichomycterus trasandianus 1	LBP19845	77964	1,603,577	9189	4314896	1,427	744	1,062,375
		Trichomycterus trasandianus 2	CZUT-IC18148	657	1,160,527	7490	3597752	1,581	678	1,071,741
Nematogenyidae	*Nematogenys*	*Nematogenys inermis*	LBP3105	3105	4,441,190	10466	5828883	1,797	816	1,465,919
Callichthyidae	*Corydoras*	*Callichthys callichthys*	LBP17423	69177	2,182,778	12,711	1,278,242	1,633	500	633,030
		Corydoras elegans	LBP57701	57701	1,833,170	14692	5762570	1,537	667	1,024,689
		Corydoras gossei	LBP544	7171	1,921,329	7580	2950751	1,268	540	685,112
	Hoplosternum	*Hoplosternum littorale*	LBP466	7282	2,602,840	9071	4892979	1,668	761	1,268,768
Scoloplacidae	*Scoloplax*	*Scoloplax dicra*	LBP11001	50522	3,626,939	9077	4897373	1,215	834	1,013,018
		Scoloplax distolothrix	LBP1938	14340	1,377,449	6233	3047277	1,164	741	862,643
Loricaridae	*Farlowella*	*Farlowella oxyrryncha*	LBP1558	11509	2,441,790	9893	4795114	1,523	769	1,171,052
	Lamontichthys	*Lamontichthys filamentosus*	LBP162	4093	1,596,203	6885	3474601	1,554	715	1,110,981
	Lasiancistrus	*Lasiancistrus saetiger*	LBP9156	42517	2,248,118	10793	5656534	1,731	835	1,444,573
Astroblepidae	*Astroblepus*	*Astroblepus grixalvii*	ANSP188920	188920	1,227,559	3666	1664830	1,008	629	633,778

Subfamily	Genus	Species	Voucher	Tissue number	Number of trimmed reads	Contigs assembled	Total bp contigs	UCE contigs	Mean length	Total bp UCES
		Astroblepus sp.	LBP19836	77952	2,309,522	5746	3219885	1,436	740	1,062,420
Characidae	*Leporinus*	*Leporinus striatus*	LBP3180	16871	3,742,443	6626	2924078	1,397	425	593,801
		Total			323,249,892	1,347,866	679,632,581	203,374	91,984	122,507,409
		average			2,099,025	8,752	4,413,199	1,321	597	795,503

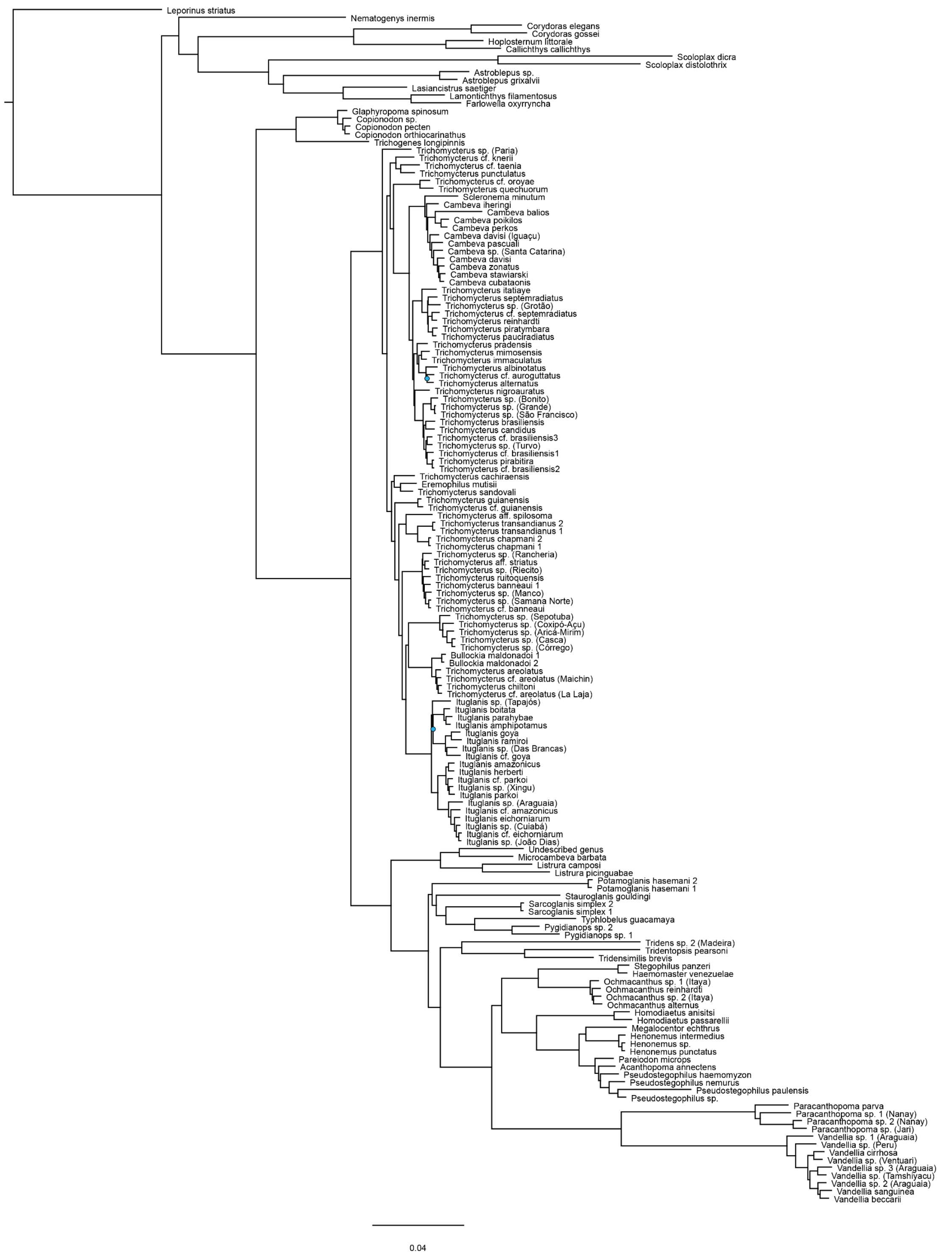

Fig 1. Bayesian inference using 50% complete matrix. Nodal support between 0.99-0.50 denoted by blue circle and support between 0.49-0.01 denoted by red circles.

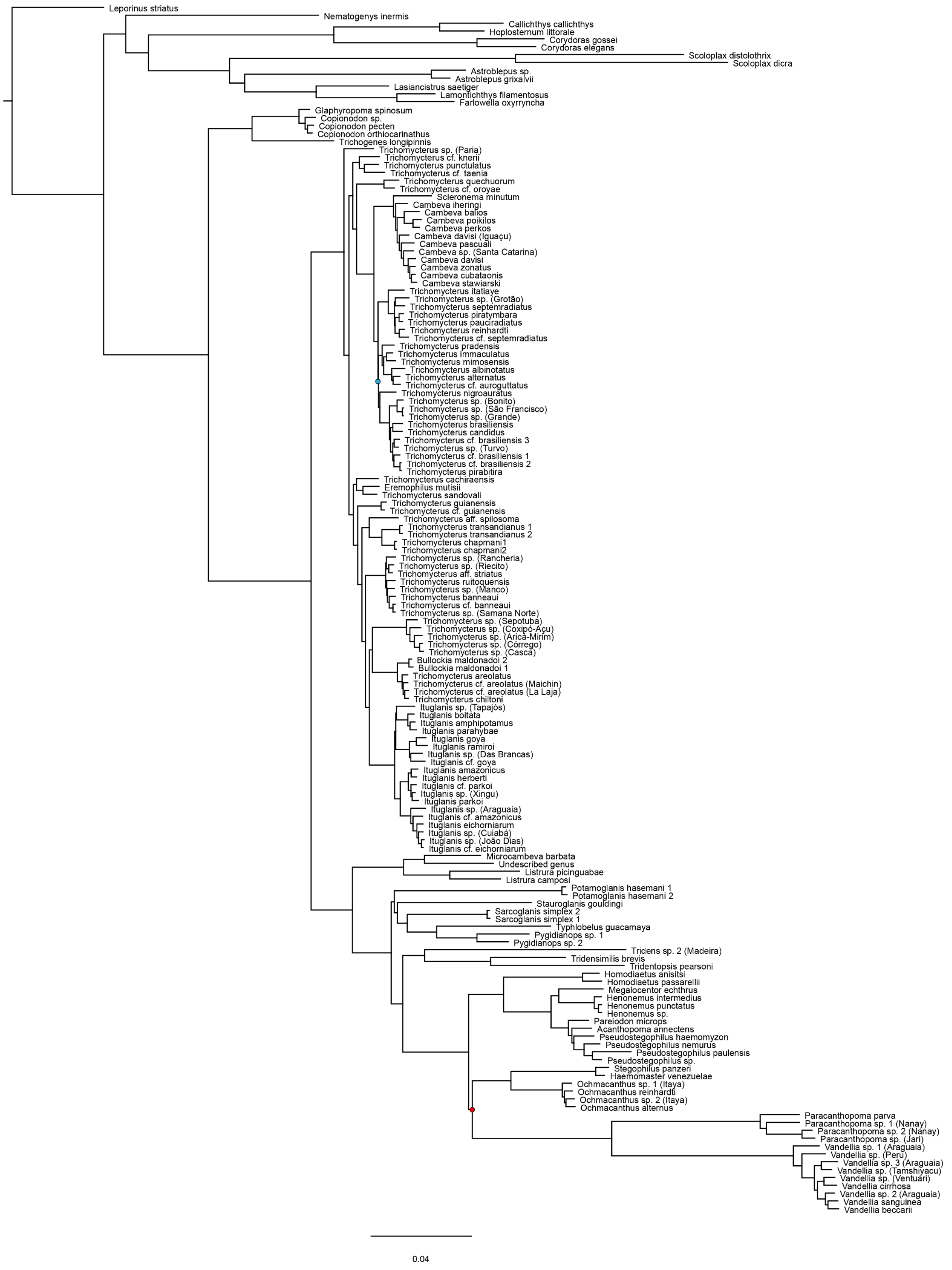

Fig 2. Bayesian inference using 90% complete matrix. Nodal support between 0.99-0.50 denoted by blue circle and support between 0.49-0.01 denoted by red circles.

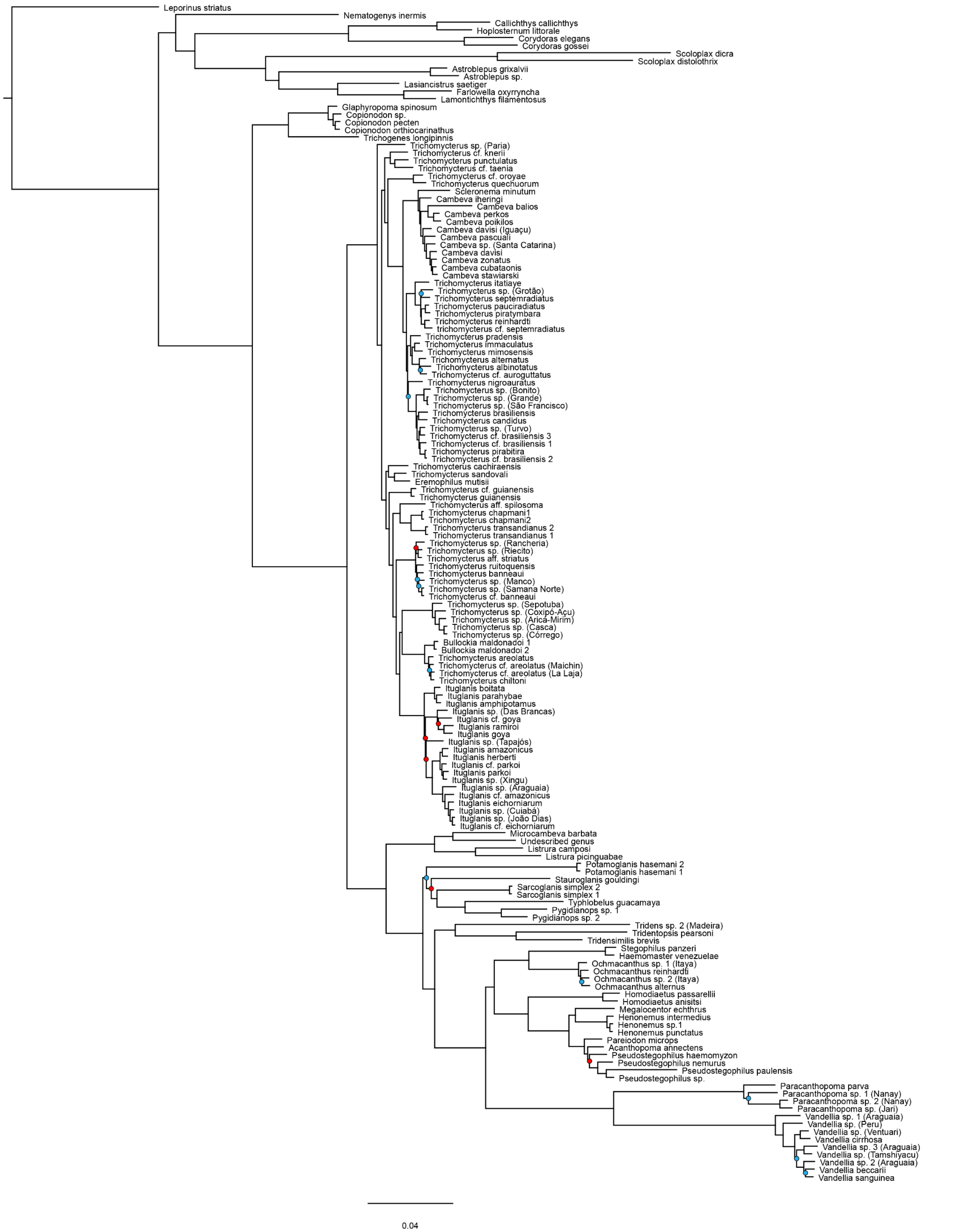

Fig 3. Maximum likelihood inference using 50% complete matrix. Nodal support between 0.99-0.50 denoted by blue circle and support between 0.49-0.01 denoted by red circles.

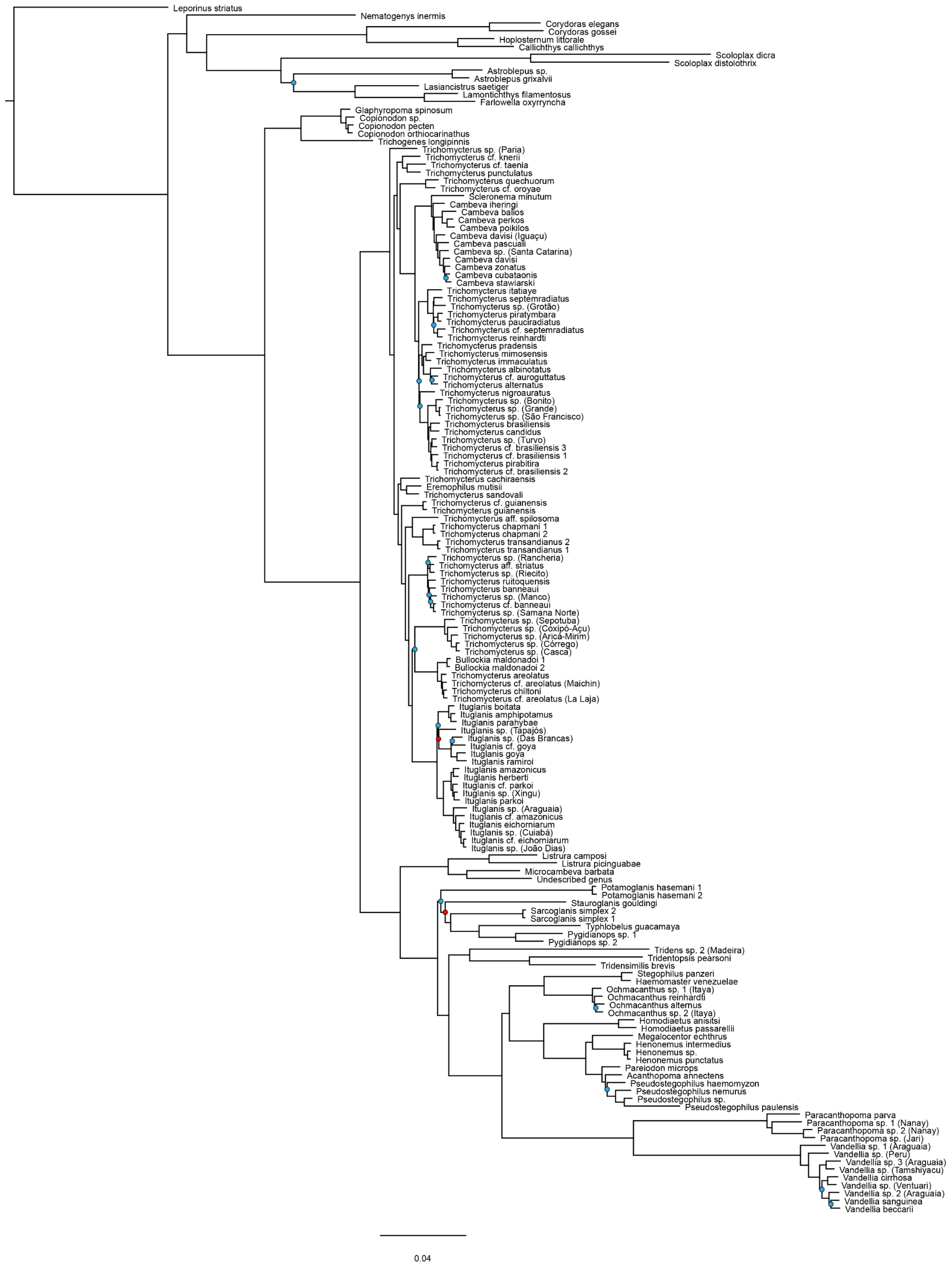

Fig 4. Maximum likelihood inference using 75% complete matrix. Nodal support between 0.99-0.50 denoted by blue circle and support between 0.49-0.01 denoted by red circles.

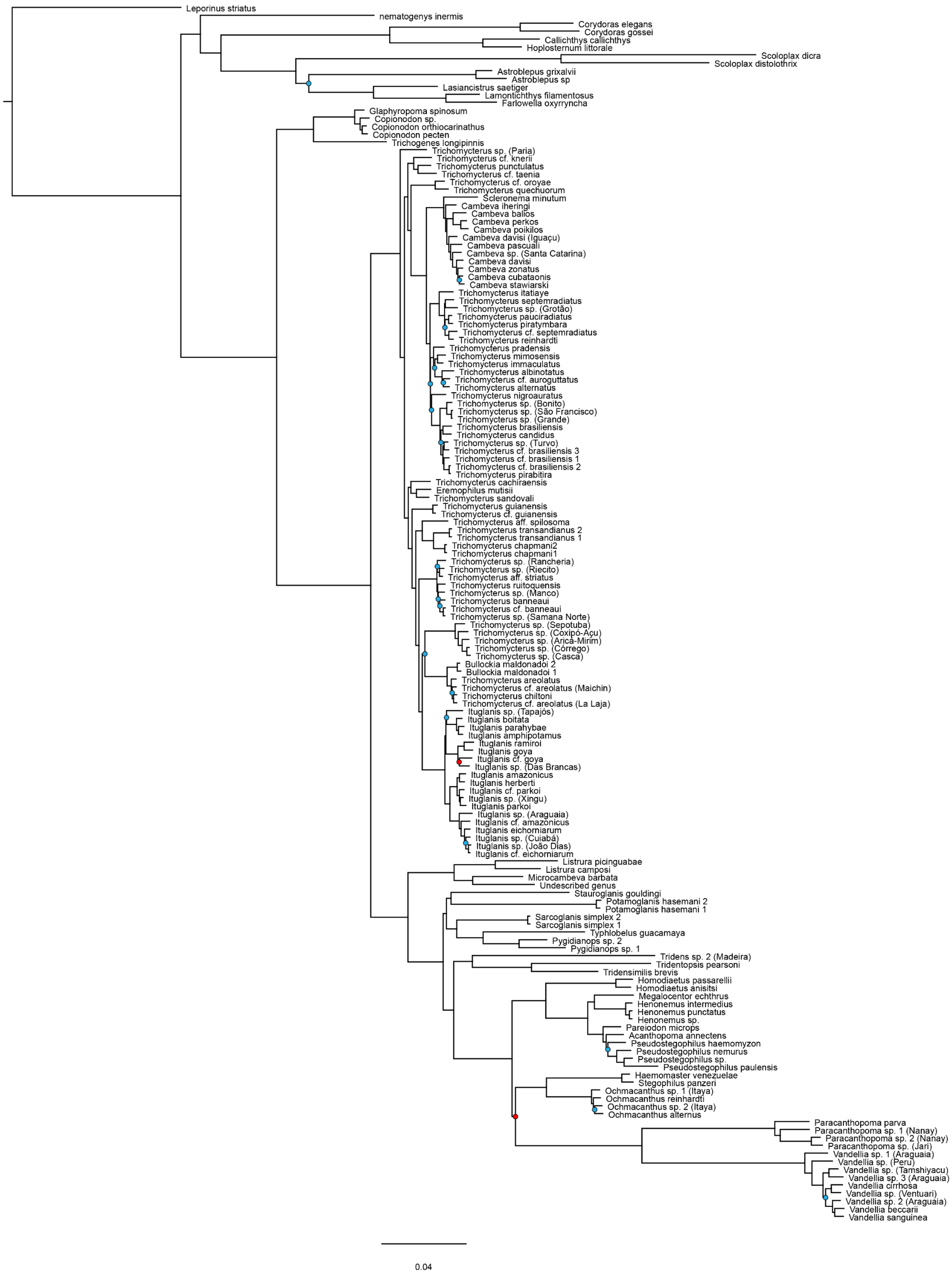

Fig 5. Maximum likelihood inference using 90% complete matrix. Nodal support between 0.99-0.50 denoted by blue circle and support between 0.49-0.01 denoted by red circles.

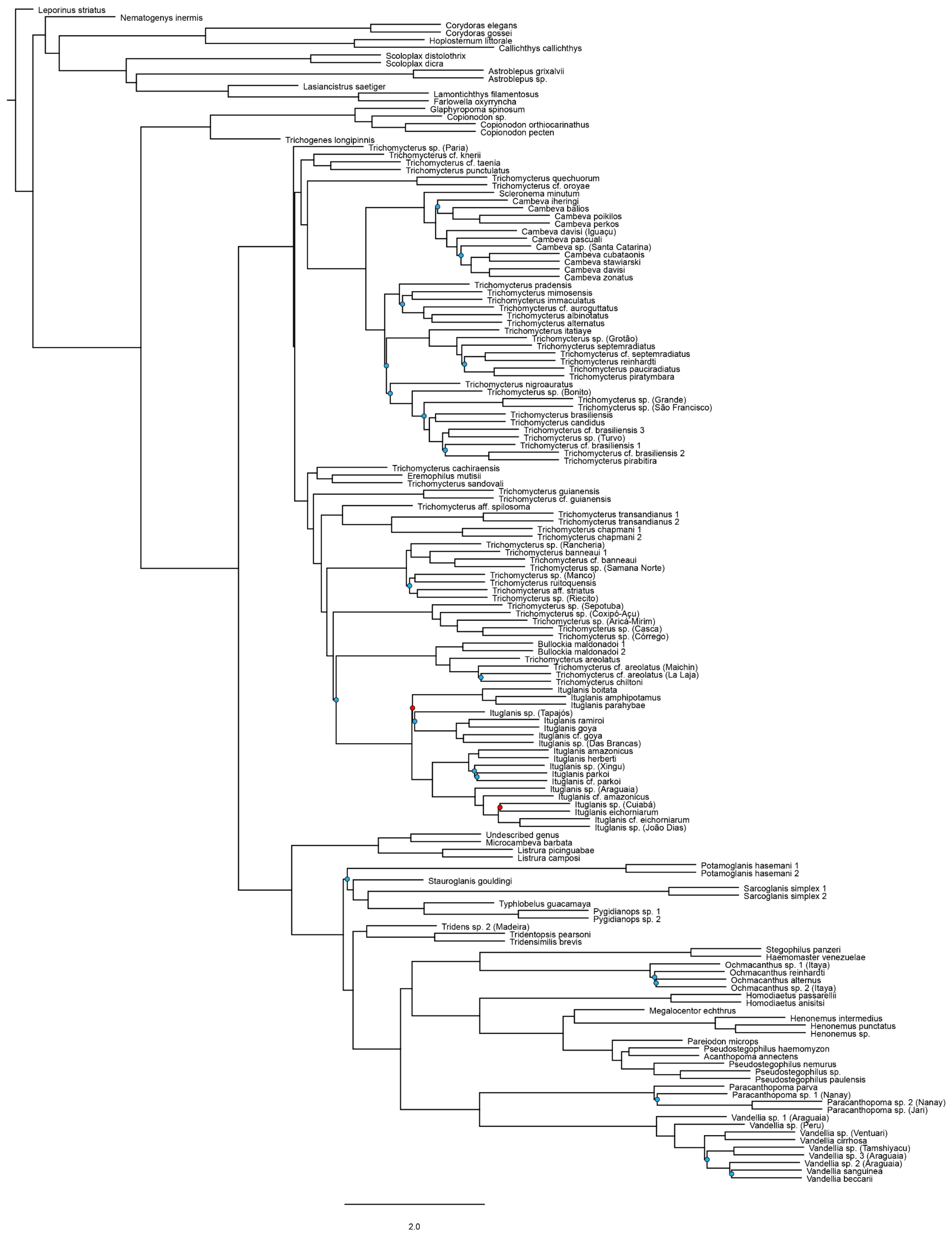

Fig 6. Species tree inference from 50% complete matrix. Nodal support between 0.99-0.50 denoted by blue circle and support between 0.49-0.01 denoted by red circles.

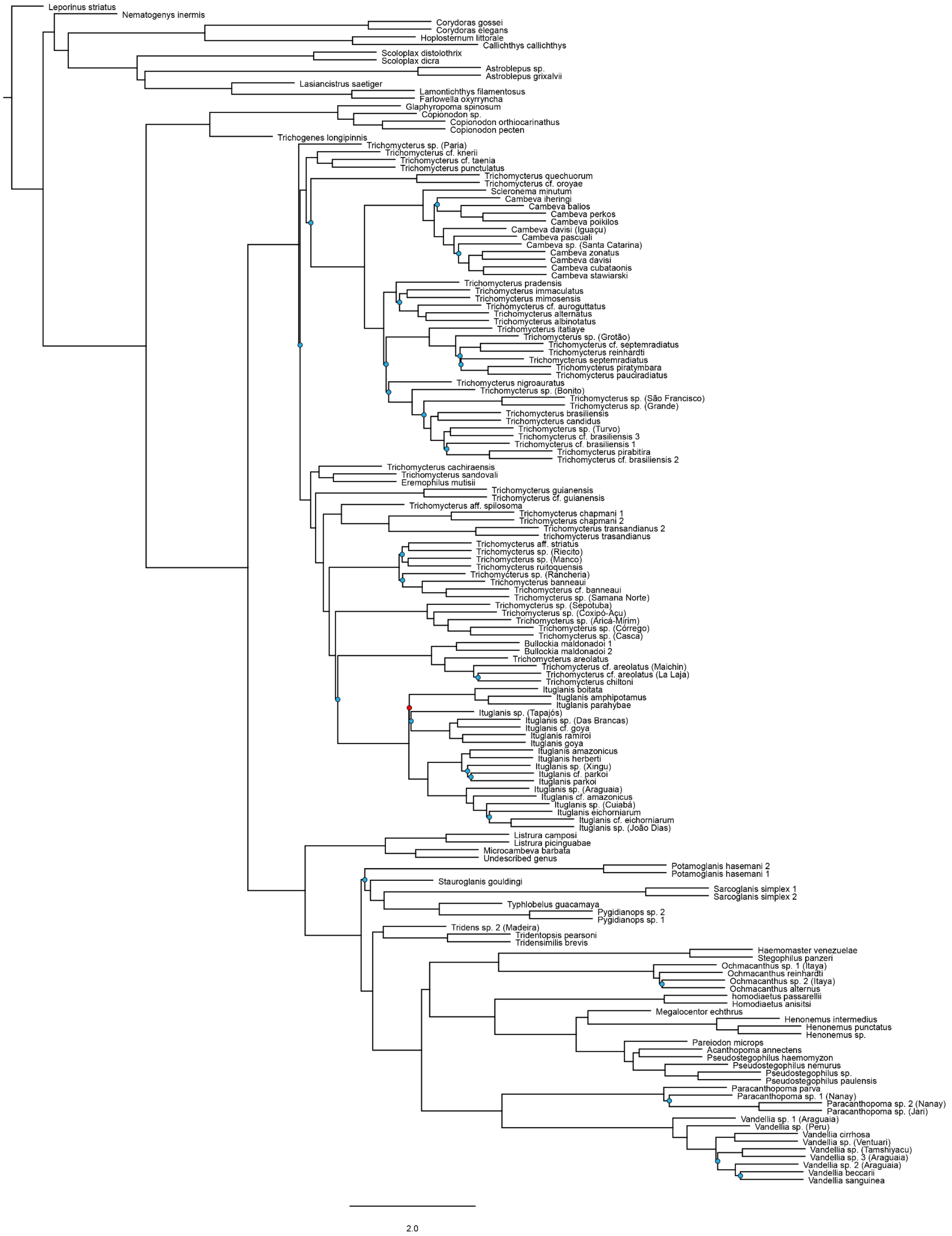

Fig 7. Species tree inference from 75% complete matrix. Nodal support between 0.99-0.50 denoted by blue circle and support between 0.49-0.01 denoted by red circles.

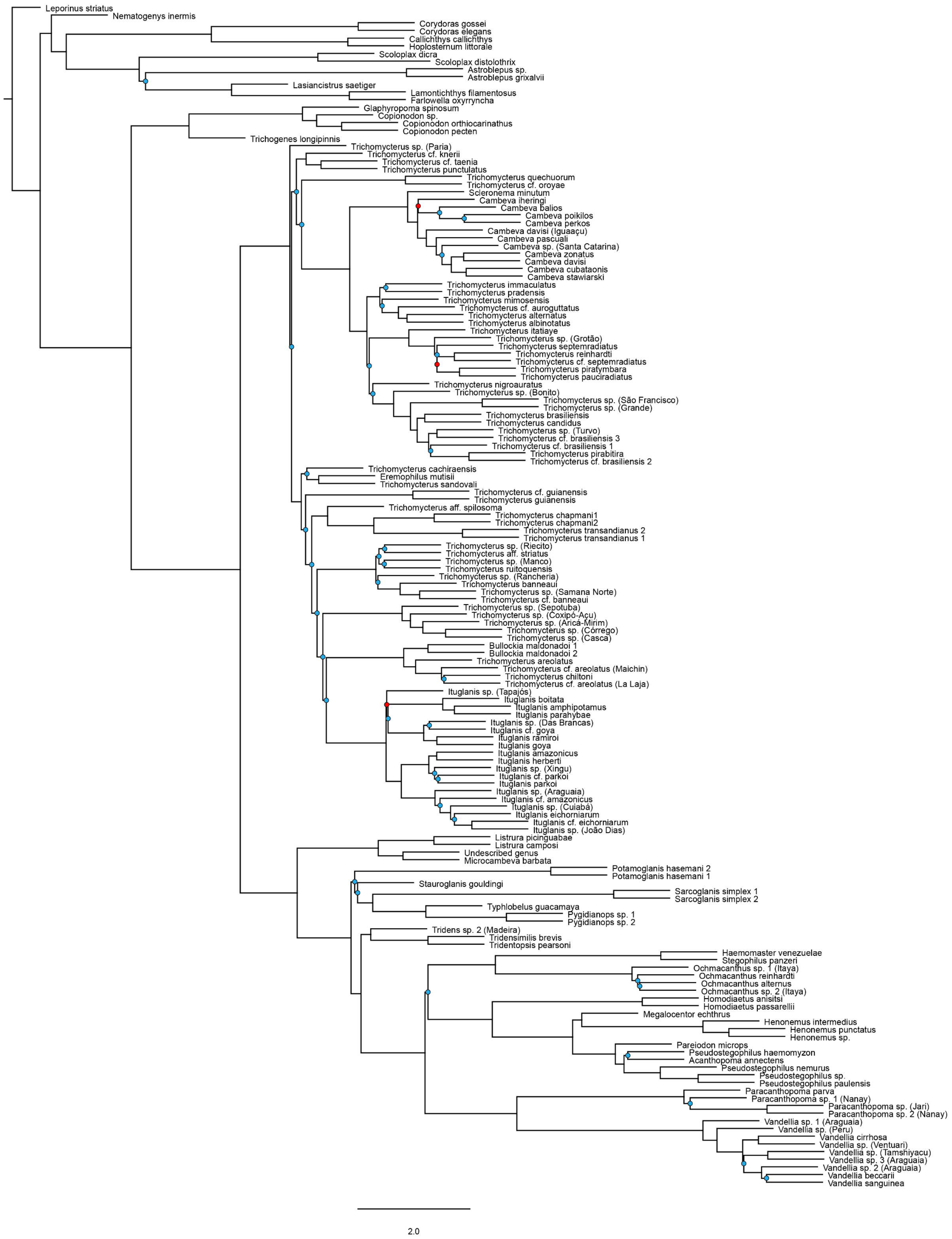

Fig 8. Species tree inference from 90% complete matrix. Nodal support between 0.99-0.50 denoted by blue circle and support between 0.49-0.01 denoted by red circles.
